# High-resolution Annual Dynamic dataset of Curve Number from 2008 to 2021 over Conterminous United States

**DOI:** 10.1038/s41597-024-03044-2

**Published:** 2024-02-15

**Authors:** Qiong Wu, John J. Ramirez Avila, Jia Yang, Cunxiong Ji, Shanmin Fang

**Affiliations:** 1 Shaanxi Key Laboratory of Earth Surface System and Environmental Carrying Capacity, College of Urban and Environmental Sciences, Northwest University, Xi’an, 710127 China; 2 Institute of Qinling Mountains, Northwest University, Xi’an, 710127 China; 3 Department of Natural Resource Ecology and Management, Oklahoma State University, Stillwater, OK 74074 USA; 4 Watersheds and Water Quality Research Lab, Richard A. Rula School of Civil and Environmental Engineering, Mississippi State University, Mississippi State, MS 39762 USA; 5 Shaanxi Water Environment Design Group Co., Ltd., Xi’an, 710021 China

**Keywords:** Hydrology, Hydrology

## Abstract

The spatial distribution and data quality of curve number (CN) values determine the performance of hydrological estimations. However, existing CN datasets are constrained by universal-applicability hypothesis, medium resolution, and imbalance between specificity CN tables to generalized land use/land cover (LULC) maps, which hinder their applicability and predictive accuracy. A new annual CN dataset named CUSCN30, featuring an enhanced resolution of 30 meters and accounting for temporal variations in climate and LULC in the continental United States (CONUS) between 2008 and 2021, was developed in this study. CUSCN30 demonstrated good performance in surface runoff estimation using CN method when compared to observed surface runoff for the selected watersheds. Compared with existing CN datasets, CUSCN30 exhibits the highest accuracy in runoff estimation for both normal and extreme rainfall events. In addition, CUSCN30, with its high spatial resolution, better captures the spatial heterogeneity of watersheds. This developed CN dataset can be used as input for hydrological models or machine learning algorithms to simulate rainfall-runoff across multiple spatiotemporal scales.

## Background and Summary

The Curve number (CN) method, initially developed in 1954 by the U.S. Soil Conservation Service (now known as USDA NRCS)^1,2^, serves as an important empirical approach to estimating surface runoff. As a derivative of the CN method, CN value is usually developed based on a combination of physical conditions, including the hydrologic soil group (HSG) and the land use/land cover (LULC) characteristics within a specific area of interest^3^. Despite the persistent doubts about the effectiveness of the CN method, CN values have gradually evolved into the primary control factor of the pervasive surface runoff simulation approach worldwide^2,4–7^.

In hydrological estimation, CN values are extensively used by hydrological models (i.e., SWAT, APEX, HEC-HMS, SWMM)^8–13^. They have proved to be an effective way of achieving satisfactory accuracy in the estimation of different hydrological processes and conditions^14–16^. Additionally, CN values now serve as crucial input variables in machine learning approaches for simulating hydrological processes^17–21^. Given that the CN value has played a significant role in various aspects of hydrological modeling for many years, the accurate determination of CN values holds great potential for enhancing the simulation accuracy of hydrological processes in the future.

Driven by the variations in soil hydrological properties, land use, agricultural practices, and antecedent rainfall conditions, CN values exhibit significant spatial variations, which further determine the distribution of runoff generation^3,22^. The spatial distribution of CN values is crucial for developing hydrological models^23^. Consequently, enhancing the spatial resolution and accuracy of the CN dataset is an effective way to improve the performance of hydrological predictions. Recently, a global Curve Number dataset (GCN250) has emerged, offering CN values at a resolution of 250 meters globally^5^. While GCN250 marks a significant advancement, its moderate resolution hinders the effectiveness of regional hydrology process simulations, particularly in small to medium size watersheds. A higher-resolution CN map, essential for detailed hydrological modeling, remains unavailable. With the release of multiperiod fine resolution and high-quality land use/land cover (LULC) products, thematic maps, and HSG databases for conterminous United States (CONUS)^24,25^, the accurate quantification of CN values in the CONUS at a higher resolution became achievable. Besides, the topographical slope, which is neglected in the derivation of CN maps^5,26^, requires to be considered for its significant potential to affect CN values^27^. To address the growing demand for accurate assessment of the spatial heterogeneity in hydrological processes, a finer resolution CN dataset that affects the quality and credibility of distributed hydrological predictions is urgently needed.

Neglecting temporal changes, even high-quality spatial CN datasets could lead to considerable errors in hydrologic responses to precipitation events, as the rainfall-runoff relationship dynamically changes over time^28–30^. Previous studies often estimated a single CN map or applied the static CN values in hydrological estimation^26,31,32^. However, the applicability of CN datasets without temporal variations has been questioned due to their universal-applicability hypothesis^2,4,33^. The static functional form of the CN dataset is considered a significant impediment to its adaptability^33^.

The temporal variation of CN patterns is controlled by antecedent runoff conditions and Land Use/Cover Change (LUCC). Several efforts have been made to develop dynamic CN values^5,34,35^. The dynamic variation of CN values caused by Antecedent Runoff Conditions (ARC) is classified as dry, average, and wet^5,34^. A dynamic CN dataset was developed by regression analysis integrated with the remotely-sensed Normalized Difference Vegetation Index (NDVI) for four small watersheds in Kansas State^35,36^. This approach is more inclined to reveal vegetation changes by adjusting CN values based on phenology, but it has failed to account for LUCC, specifically alterations in the hydrologic soil-cover complex. Given the intensification of human activities in recent years^37–39^, instances of LUCC significantly altering CN values have been scarcely reported. Therefore, it remains crucial to create a dynamic CN dataset that comprehensively captures the temporal variability resulting from both antecedent runoff conditions and LUCC.

Limitations in existing CN datasets also arise from the imbalance between detailed LULC classifications CN Tables in the National Engineering Handbook Part 630 (NEH-630)^40^ and the more generalized classifications in remote sensing LULC products (such as the European Space Agency Climate Change Initiative Land Cover Project). Therefore, this disparity introduces considerable uncertainties in existing CN datasets at both national and global levels^5,26,31,32^. With the release of more advanced land cover datasets such as the National Cropland Data Layer (CDL), the National Forest Type Dataset (NFTD), and the National Land Cover Database (NLCD) in CONUS, the precision of CN values assigned by the NEH-630 CN table is expected to significantly improve.

In this study, we developed the CUSCN30 dataset to characterize the inter-annual changes in CN values across the CONUS from 2008 to 2021. This dataset was generated at a spatial resolution of 30 m and incorporates a wide range of LULC categories derived from various advanced datasets. We also analyzed the impact of LUCC on CN values. This study provided a valuable CN dataset for the CONUS, with the primary goal of enhancing the prediction accuracy of hydrological processes and hydrologic modeling. As an essential dataset, CUSCN30 is expected to be a valuable tool for simulating hydrological processes and advancing the field of hydrology in the future.

## Methods

### Data collection

We compiled various gridded datasets in the CONUS, including LULC, HSG, and digital elevation model (DEM), to develop the CUSCN30 dataset. The LULC was from three sources: CDL, NFTD, and NLCD. Specifically, the CDL, as a crop-specific annual land cover data layer for the CONUS using moderate resolution satellite imagery and extensive agricultural ground (Boryan *et al*.) 2011^41^. The NFTD dataset, including various forest types across the CONUS, was collaboratively developed by the US Forest Service (USFS) Forest Inventory and Analysis (FIA) program and the Geospatial Technology and Applications Center (GTAC). The dataset delineates 28 distinct forest type groups within the CONUS^42^. The NLCD databases were produced by the Multi-Resolution Land Characteristics (MRLC), a group of federal agencies that coordinate and generate consistent and relevant land cover information at national scale for a wide variety of environmental, land management, and modeling applications^43^.

The HSG data, obtained from 30 m resolution Soil Survey Geographic Database (SSURGO) database, provides detailed soil information across the United States. It has been collected over a century by the National Cooperative Soil Survey partnership^44^. It is noteworthy that SSURGO has missing data in some areas within the CONUS. To address this, the database Global Hydrologic Soil Groups (HYSOGs250m) for USDA-based CN runoff modeling was resampled into 30-m grids as supplementary datasets^45^. The land slope was developed from the 30-meter DEM data from the Shuttle Radar Topography Mission (SRTM)^46^.

Furthermore, we estimated watershed-level CN value based on streamflow and precipitation datasets, compiled from the United States Geological Service (USGS) National Water Information System (NWIS)^47^. The Hydrologic Units Code 12 (HUC12) watershed boundary data was extracted from the USGS Watershed Boundary Dataset (WBD)^48^. To compare the CN results, we also acquired the GCN250 dataset from Hadi *et al*.^49^.

### CN mapping process

**Fig. 1:**
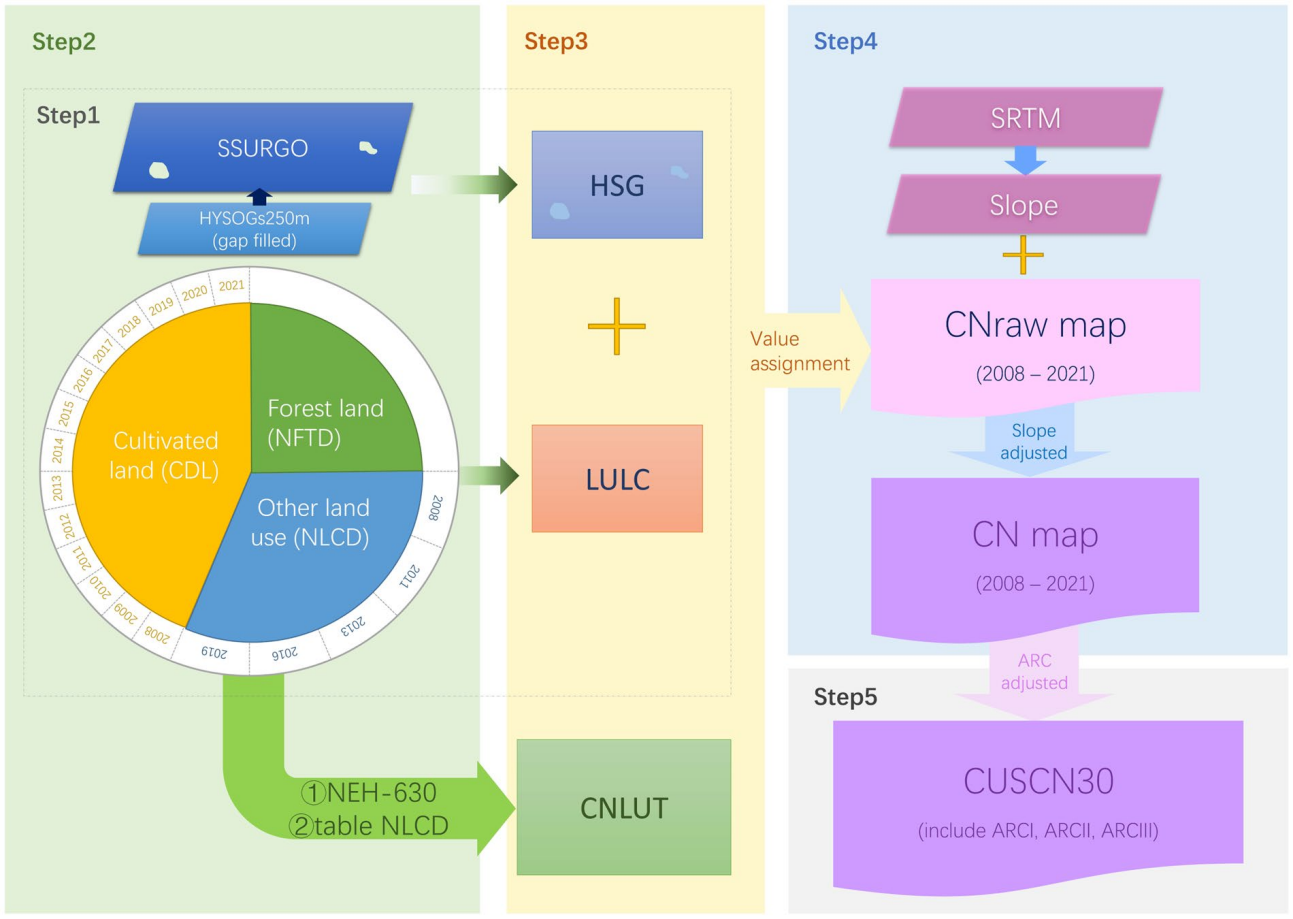
Data Processing Processes of CUSCN30.

Figure 1 illustrates the data processing scheme employed to generate the spatial CN distribution for the CONUS from 2008 to 2021. This CN mapping process involves five key steps. Prior to these processes, all datasets were preprocessed to ensure data integrity, coordinate systems uniformity, and pixel alignment.

#### Step 1: Hydrologic soil-cover complex

The annual LULC dataset was generated by overlap analysis of cultivated land types from CDL (14 scenes), NFTD (1 scene), and NLCD (5 scenes) during 2008 to 2021. To address overlapping and inconsistent types, a priority order of LULC datasets was established as CDL > NFTD > NLCD. The CDL data contains 107 types of crops, predominantly corn, soybean, fallow/idle cropland, winter wheat, and alfalfa. The NFTD includes 28 forest groups such as western white pine group, oak/pine group, maple/beech/birch group, and tropical hardwoods. Uncovered areas were filled using the land cover data in the NLCD databases. And HYSOGs250m dataset was used to fill the missing data of SSURGO database. Dual HSGs (A/D, B/D, and C/D) were assigned to hydrological group D, following the recommendations of Jaafar *et al*.^5^, Victor *et al*.^50^, and Van *et al*.^51^.

#### Step 2: CNLUT mapping

The CNLUT was created by combing the CN table from NEH-630^3^ and the NLCD^52^ table, based on the hydrologic soil-cover complex data obtained in Step 1 of the study. In accordance with the original CN tables, a wide array of specific LULC classifications were consolidated into 28 representative types. The combination of these LULC representative types and 4 HSGs (HSGs: A/B/C/D) resulted in a total of 112 distinct hydrologic soil-cover complex classes (Table 1). This table also provides a comprehensive overview of the CN values associated with each hydrologic soil-cover complex.

**Table 1:** The specific information of CNLUT, including LULC, HSG, and the referred CN values (adapted from NEH-630 and table NLCD).

LULC	HSG			
	A	B	C	D
Fair row crops	70	80	87	90
Good row crops mix water	85	90	93	95
Fair CBR	62	75	83	87
Fair small grain	64	76	84	88
Fair CBR mix fair small grain	63	75	84	88
Good meadow	30	58	71	78
Fallow bare soil	77	86	91	94
Open Water	100	100	100	100
Perennial ice/snow	100	100	100	100
Developed, open space	45	65	76	82
Developed, low intensity	60	74	82	86
Developed, medium intensity	77	85	90	92
Developed, high intensity	92	94	96	96
Barren land	77	86	91	94
Brush mixture	48	67	77	83
Pasture	49	69	79	84
Woody wetlands	78	78	78	78
Emergent herbaceous wetlands	85	85	85	85
Poor row crops	72	81	88	91
Fair row crops C	68	77	83	87
Poor row crops mix fair small grain SR	67	78	85	89
Poor row crops mix fair CBR	66	77	85	89
Fairly good wood	33	58	72	78
Poor wood	45	66	77	83
Fair wood	36	60	73	79
Good wood	30	55	70	77

Note: CBR means close-seeded or broadcast legumes or rotation meadow; crop C means the contoured crop; small grain SR means straight row of small grain.

#### Step 3: Assigning CN values

In order to create the annual CN maps across CONUS from 2008 to 2021, CN values were assigned to the annual hydrologic soil-cover complexes using references from the CNLUT developed in Step 2. It is worth noting that despite the presence of 112 categories of hydrologic soil-cover complexes, only 46 unique CN values (CNraw) were derived from the CNLUT due to the duplicated values, as indicated in Table 1. This CN value assignment process ensured that the annual LUCC was thoroughly considered and accurately reflected in the CN values.

#### Step 4: Slope-adjusted

Given the potential influence of slope on CN values in finer resolution mapping. Therefore, we employed a slope-adjusted formulation of CN values (CN_slope_), to incorporate terrain variations^53^. The equation is expressed as:

$$C{N}_{slope}=C{N}_{raw}\frac{322.79+15.63\left(\alpha \right)}{\alpha +323.52}$$

where the slope *α* (m/m) is considered valid within the range of 0.14 to 1.4 to remain consistent with experimental values. It’s important to note that while *CN*_*slope*_ values should never exceed 100, CN values for open water, initially set at 100 for all HSG types, exceeded this limit when the slope *α* exceeded 0.05. To address this issue, any CN values exceeding the limit were adjusted down to 100.

#### Step 5: ARC

To accommodate climate variability and seasonal fluctuations, we developed three ARC scenarios: dry (ARC-I), average (ARC-II), and wet (ARC-III). We utilized the cumulative distributions of CN values for different ARCs: 10% for ARC I, 50% for ARC II, and 90% for ARC III, following the Grabau *et al*.^54^ and Donald *et al*.^40^. The CN values for the ARC-I (*CN*_ARCI_) and the ARC-III (*CN*_ARCIII_) scenarios were calculated based on the CN values established for ARC-II (*CN*_ARCII_, following the methodology outlined in refs. ^12,55^.

$$C{N}_{{\rm{ARCII}}}=C{N}_{slope}$$

$$C{N}_{{\rm{ARCI}}}=C{N}_{{\rm{ARCII}}}-\frac{20\times \left(100-C{N}_{{\rm{ARCII}}}\right)}{100-C{N}_{{\rm{ARCII}}}+\exp \left(2.533-0.0636\times \left(100-C{N}_{{\rm{ARCII}}}\right)\right)}$$

$$C{N}_{{\rm{ARCIII}}}=C{N}_{{\rm{ARCII}}}\times \exp \left(0.00636\times \left(100-C{N}_{{\rm{ARCII}}}\right)\right)$$

### Validation

**Fig. 2:**
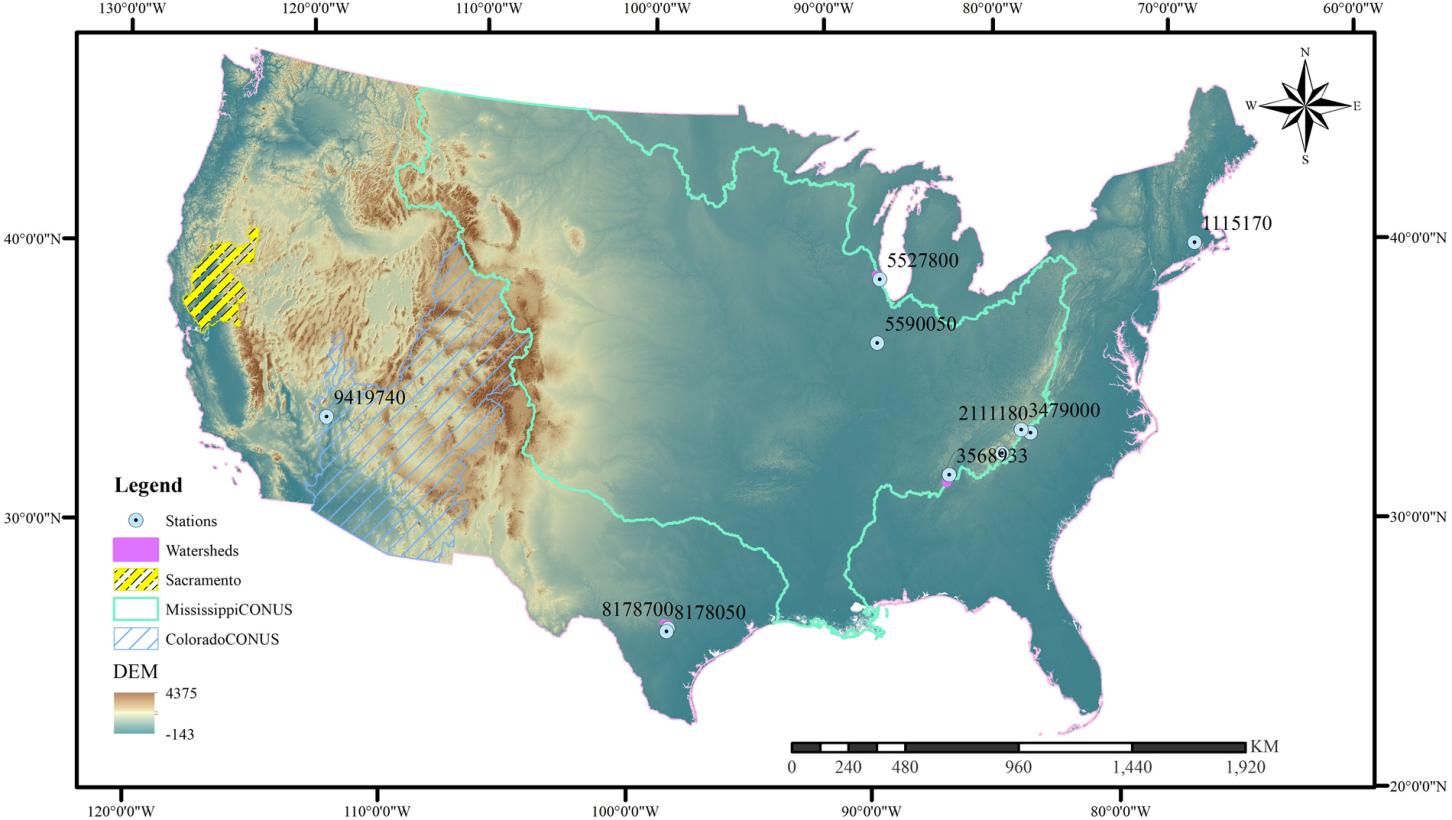
Study areas of the Continental United States and the selected watersheds and USGS stations to validate the developed curve number data.

The ‘observed’ surface flow is calculated using the USGS Groundwater Toolbox, based on the NWIS streamflow and precipitation dataset from 2008 to 2021. Specifically, the hydrograph separation methods PART and HySEP (including HySEP-Fixed, HySEP-Slide, and HySEP-LocMin methods) are employed. Watershed boundaries were aligned with HUC12 data, and any sites not conforming to the HUC12 boundary were delineated using ArcGIS Hydrology Tools based on 30 m SRTM data. The area of all watersheds is required to be within an area range of 1 to 500 mi^2^, due to the limitation of the baseflow separation procedure^56^. The 10 watersheds represent a wide geographic and climate distribution within the CONUS (Supplementary Table 1 and Fig. 2) and were selected for validating our developed CN data.

To get the watershed CN value, we employed the rainfall-runoff relationship method as described by Donald *et al*.^40^. For each event rainfall P, the general conservation of mass statement for a rainstorm is:

$$P={I}_{a}+F+Q$$

Where *P* is the rainfall depth (mm), *I*_*a*_ is the initial abstraction of the rainfall (mm), *F* is the cumulative infiltration excluding *I*_*a*_ (mm), and *Q* is the surface runoff (mm). To establish a proportionality between the runoff to rainfall depths ratio and the infiltration depth to potential abstraction ratio, the equation is converted into:

$$\frac{Q}{P-{I}_{a}}=\frac{F}{S}$$

$${I}_{a}=\lambda S$$

Where *λ* is the initial abstraction ratio, set as 0.2 for this study; *S* is the potential maximum retention or infiltration (mm) according to *λ*. The original formula is defined as follows:

$$Q=\frac{{(P-0.2S)}^{2}}{P+0.8S}\,for\;P\ge 0.2S$$

$$Q=0\quad \quad for\;P < 0.2S$$

Here, *S* is obtained by the following equation:

$$S=5\left(P+2Q-{\left(4{Q}^{2}+5PQ\right)}^{1/2}\right)$$

and *CN* represents the ‘observed’ CN value at the watershed scale, calculated as:

$$CN=25400/\left(254+S\right)$$

Subsequently, we calculated the average CN for each watershed by aggregating the pixel-level CN value from our developed dataset. This ‘observed’ CN was then compared with CUSCN30 data to assess the accuracy.

## Data Records

The CUSCN30 dataset between 2008 and 2021 is available at Zenodo^57^ (10.5281/zenodo.10474320). It’s important to note that the CUSCN30 dataset is published as a fully open dataset with CC-BY licenses.

Each zip file contains the CN data for a specific year at a 30-m spatial resolution in TIF format. To access the data for a particular year, you can download the corresponding zip file and use unzip software as needed.

## Technical Validation

### Comparison between CUSCN30 with measurements

#### CN values

To illustrate the accuracy of CUSCN30, we compared the CN values derived from observed CN, CUSCN30, and GCN250 datasets across 10 small watersheds in the CONUS. The average of observed CN values, obtained from 4 different methods (PART, fixed-interval HySEP-Fixed, sliding-interval HySEP-Slide, and local minimum HySEP-LocMin), are presented in Supplementary Table 2. The variance in observed CN among these methods at each site ranged from 0.01 to 1.45, indicating a strong consistency in the observed CN estimates derived from the NWIS dataset.

**Fig. 3:**
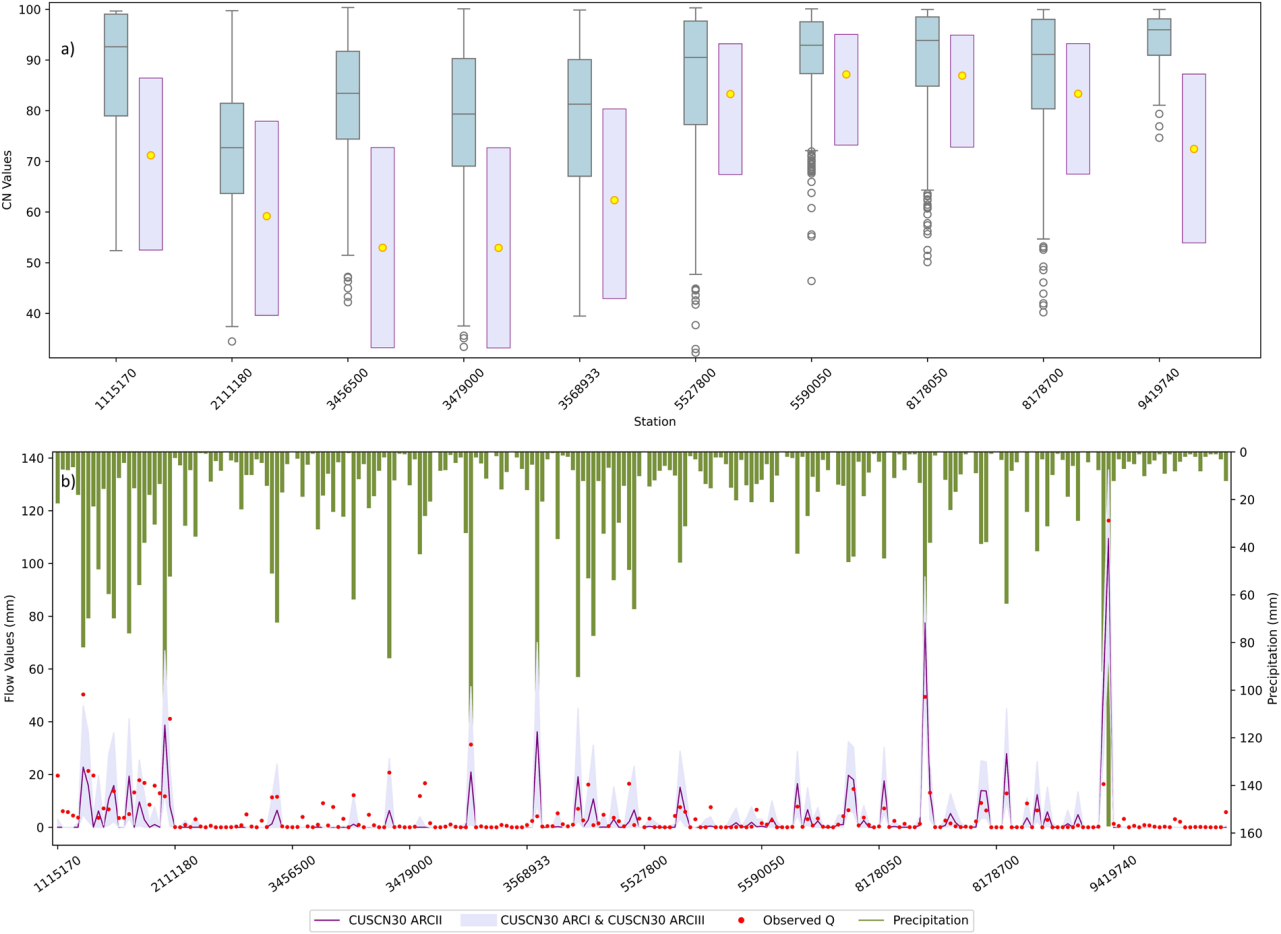
Observed and CUSCN30 estimated values from the 10 watersheds: (**a**) the blue boxplots of observed curve number (CN) and estimated CN values of CUSCN30 in dry, average, and wet, (**b**) estimated surface flow, observed surface flow, and precipitation for the random selected events.

Generally, the CN values obtained from CUSCN30 were lower compared to observed CN (Fig. 3a,b), indicating an underestimation of CN values. This finding is consistent with the previous studies^32^. For stations 1115170, 8178050, 8178700, 5527800, and 8178700, CUSCN30 provided accurate CN values, as observed CN values fell within the range of wet (ARCIII) to dry (ARCI). However, the other sites demonstrated lower estimations compared to the observed CN. The underestimation of CUSCN30 CN against observed CN can be attributed to the lower CN value associated with ‘Fairly good wood’ in the modified Curve Number look-up table (CNLUT) as Table 1. Similar CN underestimations in forestland areas have also been reported by Tedela *et al*.^58^ in Eastern United States, and by Lal *et al*.^59^ in India. Furthermore, as reported by Donald *et al*.^40^, the application of the CN method in forested areas characterized by HSG A, B, and C is a matter of concern.

#### River flow

The observed Q value and estimated Q based on CUSCN30 are shown in Fig. 3b. The analysis involved a dataset of 23 randomly chosen events for each station. Most of observed Q values fall within the range encompassed by CUSCN30 ARCI to ARCIII. For lower observed Q values, the estimated Q values closely align with the observed Q values, indicating a better simulation performance. Conversely, for higher observed Q values, the CN simulation displays more pronounced variability across events. But these variations typically fall within the area of ARCI and ARCIII.

Similar to the result of observed CN values, the Q values from stations 1115170, 8178050, 8178700, 5527800, and 8178700 showed a high level of accuracy. Conversely, watersheds with underestimated CN values, such as stations 2111180, 3479000, 2111180, and 9419740, are more likely to exhibit underestimated runoff. Notably, these sites are predominantly covered by ‘Fairly good wood’, with land cover percentages ranging from 66.9% to 90.1% (Supplementary Table 1).

### Comparison between CUSCN30 and other CN datasets

#### Data resources and methodologies

Table 2 shows the basic information of three CN datasets: Zeng^26^, GCN250, and our CUSCN30, which are used for comparative analysis in this study. For input HSG data, the Harmonized World Soil Database (HWSD) used by Zeng combines regional and national updates of soil information worldwide with the content from the FAO-UNESCO Soil Map of the World. And HYSOGs250m dataset employed in the GCN250 CN datasets was generated using USDA-based soil texture classes, depth to bedrock, and depth to groundwater table^60^. In contrast, the Soil Survey Geographic Database (SSURGO) was gathered by direct field observations^61^.

**Table 2:** Basic information of three CN datasets.

Zeng CN		GCN250	CUSCN30	
Basic information	—		Available online	
	Global		CONUS	
	Not mentioned	250 m	30 m	
	ARCII	ARCI, ARCII, ARCIII		
	Single temporal (2013)	Single temporal (2015)	14 annual (from 2008 to2021) datasets that consider the LUCC of each year	
Input Data	HSG	The Harmonized World Soil Database (HWSD) v1.2 (1000 m)	HYSOGs250 (250 m)	SSURGO (domain, 30 m) and HYSOGs250 (fill gap, 250 m)
	LULC	The Land Cover Yearly L3 Global 500 m (MCD 12Q1) of 2013	ESA CCI-LC 2015 (300 m)	Overlap from CDL (30 m), NFTD (250 m), and NLCD (30 m)
Methodology	CN Table	Proposed by Hong and Adler (2008)	Part 630 Hydrology land cover classes (NEH-630)	Part 630 Runoff curve numbers for urban areas, cultivated agricultural lands, other agricultural lands (NEH-630), CN for NLCD
	ARC	—	Proposed by Hjelmfelt (1991)	Proposed by Arnold (1994) and Arnold *et al*. (1990)
	Slope Adjusted	—	Proposed by Huang *et al*. (2006)	

As to input LULC data, the MCD 12Q1 is obtained from the Moderate Resolution Imaging Spectroradiometer (MODIS) satellite, and ESA CCI-LC used in GCN250 provided by the European Space Agency’s (ESA) Climate Change Initiative (CCI). The CDL and NLCD are derived from the Landsat satellite^62,63^. However, it’s important to note that the LULC data overlap from CDL, NFTD, and NLCD, which offer a higher resolution, is limited to the U.S.

In terms of CN table mapping, the lookup table from Zeng was generated based on the CN lookup tables from the USDA handbook and National Engineering Handbook Section 4^3,64^. Notably, NEH-4, which underwent an update in 2004 and evolved into NEH-630, has been embraced by both GCN250 and CUSCN30 models. Owing to its more detailed LULC classifications, CUSCN30 incorporates an additional CN lookup table specifically for NLCD. The improved consistency between the CN lookup tables and LULC within CUSCN30 potentially enhances its accuracy. While the ARC methodology proposed by Hjelmfelt *et al*.^65^ is a component of NEH-630, the version developed by Arnold *et al*.^12^ and Loucks *et al*.^55^ see broader use in the Soil and Water Assessment Tool. In addition, CUSCN30 accounts for slope adjustment and LUCC, further enhancing its applicability.

#### CN values of watersheds

The CN value of the CUSCN30 was compared with the previous global CN dataset developed by Zeng *et al*. (2017) and GCN250^5^ as reported in Table 3. Only three basins were selected due to the unpublished CN map from Zeng. However, the Mississippi and Colorado river basins were partially excluded, covering 98.80% and 91.45% of their total areas respectively, due to the constraints of the CONUS boundary. The CUSCN30 CN value for the Sacramento River Basin was marginally lower by 0.81% and 1.08% compared to CN values from Zeng and GCN250. This discrepancy primarily results from the differences in the LULC and HSG datasets. The CUSCN30 CN values for the Mississippi and Colorado river basins were 4.97% and 10.79% higher than those in the study of Zeng *et al*.^26^ but 1.37% to 1.84% lower than those in GCN250, respectively. The differences in the CN values for dry and wet conditions between CUSCN30 and GCN250 are attributed to the different retrieved tables^8,40^.

**Table 3:** The average CN value of Mississippi, Colorado, and Sacramento basins in 3 CN datasets.

Watershed	Proportion	Zeng	GCN250			CUSCN30		
		CN	CN (dry)	CN (average)	CN (wet)	CN (dry)	CN (average)	CN (wet)
Mississippi	98.80%	**72**	59.3	**77**	89.3	57.55	**75.58**	89.08
Colorado	91.45%	**66.5**	56.5	**74.7**	87.6	55.32	**73.68**	87.96
Sacramento	100.00%	**74.1**	56.2	**74.3**	87.4	55.12	**73.51**	87.85

### Difference between CUSCN30 dataset and GCN250

#### Predicted runoff

**Fig. 4:**
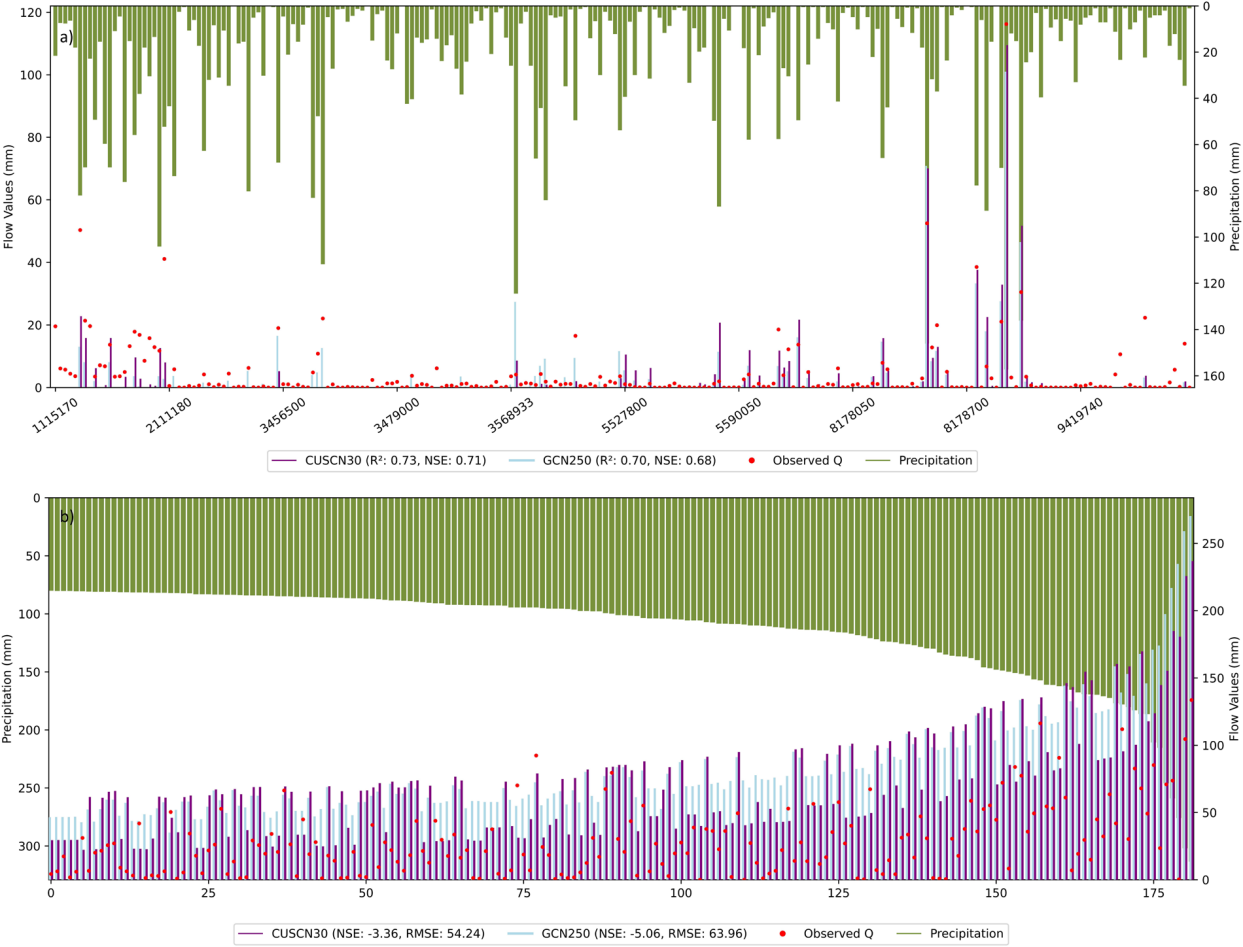
Comparison of the estimated surface flow from CUSCN30 and GCN250 data with observed surface flow: (**a**) normal precipitation events based on randomly selected. (**b**) extremely precipitation events, when precipitation exceeds 80 mm.

To investigate the differences in CN values between CUSCN30 and GCN250 datasets, Fig. 4a shows a comparison of estimated Q values. These estimated Q values are calculated based on the respective CN values from each dataset under their corresponding ARC. A total of 230 events were selected for the analysis, which was conducted on a set of random selected with 23 events for each station. These events offer a comprehensive view of hydrological dynamics, covering a spectrum of observed Q values from approximately 0 to 120 mm and simulated Q values ranging up to 160 mm. For CUSCN30, the R² value was 0.73, with an F-statistic of 629.30, and the Nash-Sutcliffe Efficiency (NSE) was 0.71. In comparison, for GCN250, the R² value was 0.70, with an F-statistic of 538.13, and an NSE of 0.68. The result indicated that CUSCN30 dataset slightly outperforms GCN250 in Q prediction.

To analyze the estimated Q values from CUSCN30 and GCN250 data under extreme rainfall events, all records that P exceed 80 mm from 10 watersheds are shown in Fig. 4b. Generally, the simulated Q is higher than the observed Q values over these events. As P increases, there is a corresponding rise in observed Q, but the increase in the estimated Q from CUSCN30 and GCN250 is more pronounced. Notably, the estimated Q from GCN250 significantly exceeds that from CUSCN30, suggesting that CUSCN30 provides more accurate estimates. Additionally, the NSE improved from −5.06 to −3.34, and the Root Mean Square Error (RMSE) decreased from 63.96 to 54.24 when using the CUSCN30 dataset. Hence, CUSCN30 demonstrated an improved performance in simulating river flow during extreme rainfall events.

#### Spatial pattern

**Fig. 5:**
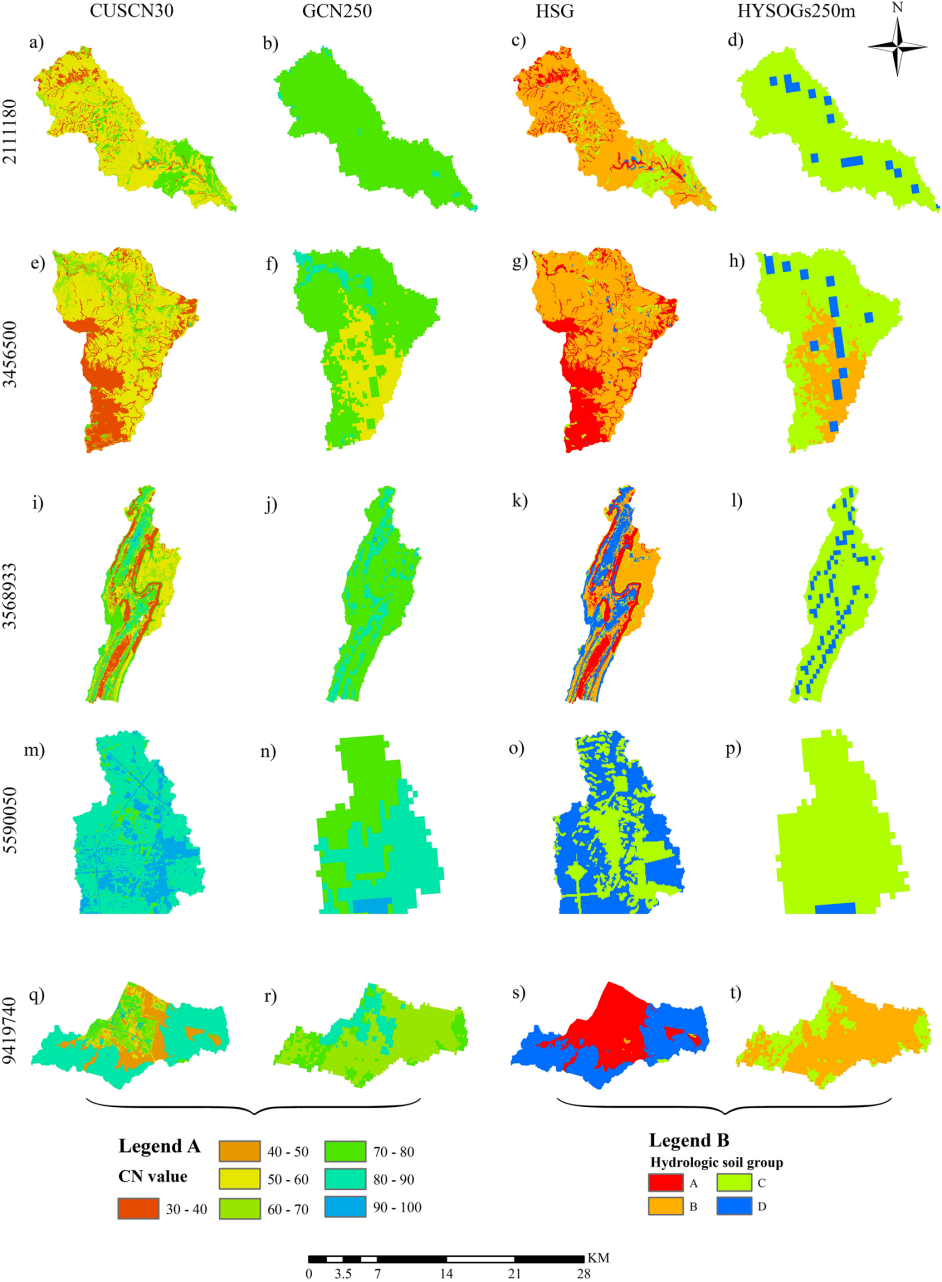
The CN values of CUSCN30 and GCN250 (Legend A) with source dataset of SSURGO and HYSOGs250m (Legend B) from 5 watersheds.

The CUSCN30 dataset provides a higher spatial resolution compared to the GCN250 (Fig. 5). Notably, the CN values showed a significant disparity between the two datasets in watershed 3456500 (Fig. 5e,f). In the CUSCN30 dataset, CN values in the southwest part of the watershed 3456500 were dominated ranging from 30 to 40. In contrast to the GCN250 dataset, these values were considerably higher, ranging from 50 to 60. Watersheds 2111180 and 3568933 exhibited a similar pattern; in the CUSCN30 dataset, CN values spanned from 30 to 70, whereas in GCN250, they ranged from 70 to 80. In contrast, in watershed 9419740, the CN values in CUSCN30 are higher than those in GCN250.

The primary cause of the discrepancy between the two CN datasets is attributable to differences in the HSG dataset. SSURGO data are presented in Fig. 5c,g,k,o,s, and HYSOGs250m data in Fig. 5d,h,l,p,t. There is a significant contrast evident between these two datasets. In the case of watershed 5590050, SSURGO identified half of the area as HSG D, whereas HYSOGs250m classified the same area into group C. Similarly, in watershed 9419740, HSGs A and D predominated in CUSCN30, in contrast to HYSOGs250m which classified it as HSGs B and C. The minimum CN value linked to HSG A was offset by the maximum value attributed to HSG D, resulting in a small difference in the average values between the two datasets. In terms of CN value ranges, SSURGO demonstrated a broader diversity in HSGs (A, B, C, D) compared to HYSOGs250m (dominated by HSGs B, C).

### Temporal and Spatial variations of CN from 2008 to 2021

#### CN variation across CONUS

**Fig. 6:**
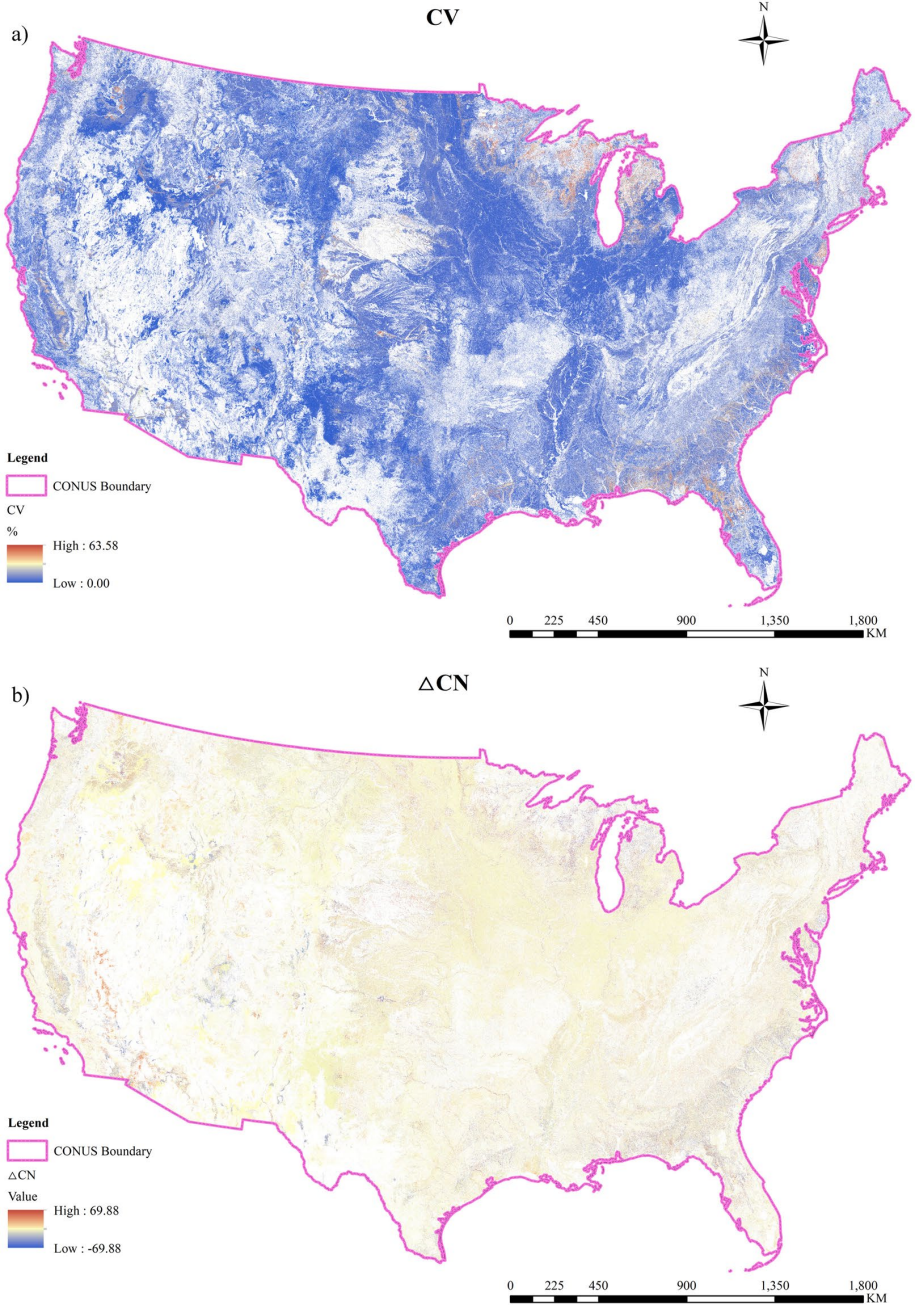
The variance of Curve Number (CN) in CUSCN30 dataset from 2008 to 2021: (**a**) CV, (**b**) △CN.

To illustrate the variation of the CN map across the CONUS, the coefficient of variation (CV) and ΔCN was used to highlight the changes in CN between 2008 and 2021. Figure 6a showed that only 46.67% of the area remained unchanged (blank area), while CV in the other areas ranged from 0.00% to 63.58%. Most of the changed areas (72.6%) have a small change with CV in the range between 0.00% to 5%. The maximum CV is 63.58%. The areas with CV in the ranges of 5–10%, 10–20%, 20–30%, 30–40%, 40–50%, and 50–63.58% represent 17.24%, 6.98%, 1.35%, 1.32%, 0.50% and 0.01%, respectively, of the study domain CV varies between regions due to the different intensities of LUCC. The area close to the Great Lakes and the coastal zone of the CONUS experienced significant changes, while the central area exhibited relatively minor alterations.

The spatial distribution of ΔCN for the CUSCN30 is displayed in Fig. 6b. Calculations were based on data from only the initial and final years, revealing that 53.33% of CN values over CONUS have changed. The average CN of the CUSCN30 shows an insignificant increasing trend (0.10/10a) from 2008 to 2021, while in the majority of the area (84.3%), the average CN showed a decreasing trend. The majority of ΔCN values fell within the range of −5 to 0, accounting for 76.8% of the CONUS. Other ΔCN ranges, including −10 to −5, −10 to −5, −20 to −10, and −69.88 to −20, represent 4.35%, 2.52%, 0.52%, and 0.18% of the CONUS, respectively. CN values increased in only 15.67% of the CONUS area, however, exhibiting a larger variation than the decreased area.

#### The impact of LUCC on the variation of CN

**Fig. 7:**
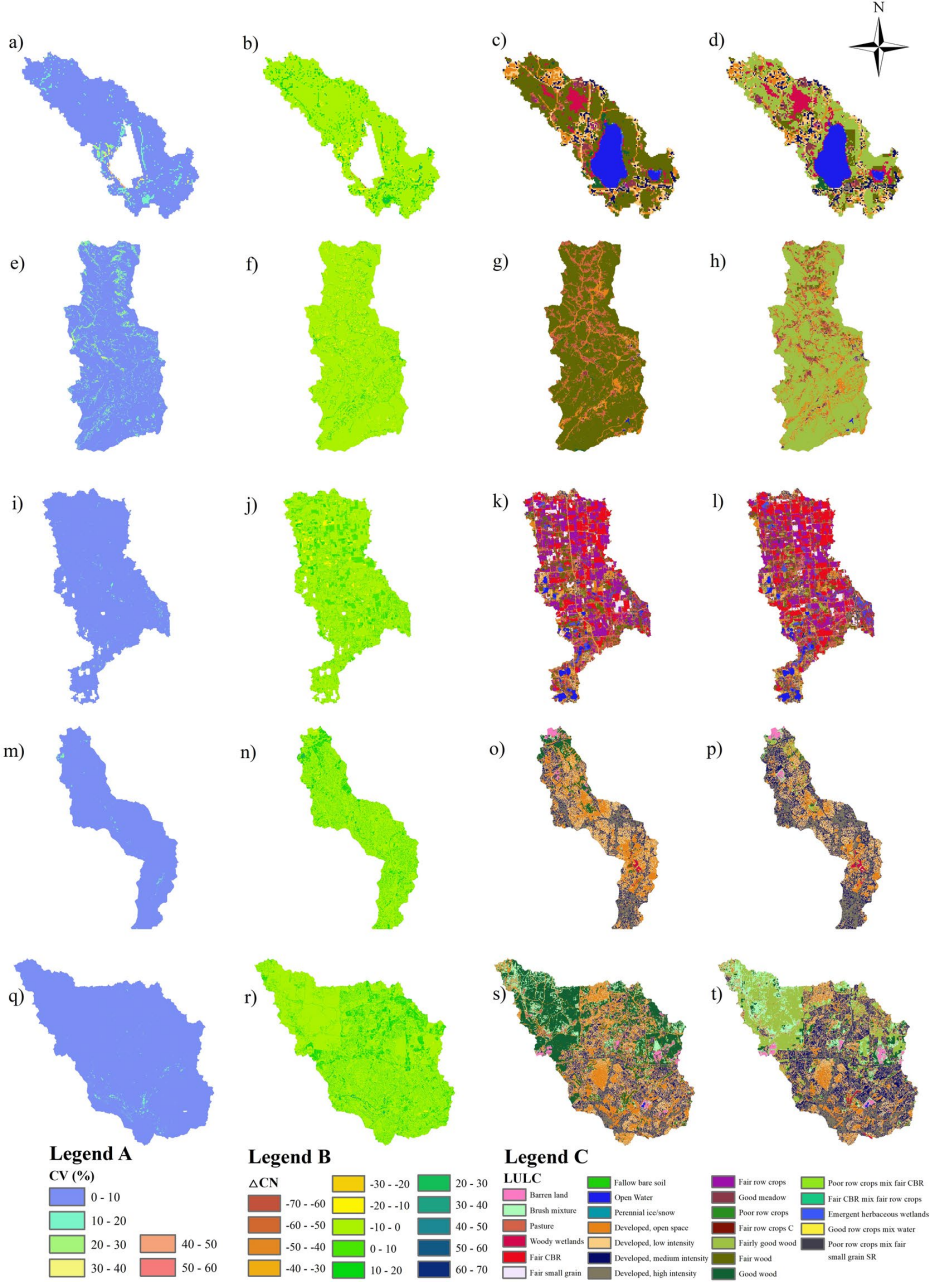
The CV (Legend A), △CN (Legend B) with source LULC datasets of CUSCN30 and GCN250 (Legend C) from 5 watersheds.

Figure 7 shows the CV, ΔCN, and different source LULC datasets of CUSCN30 for 2008 and 2021 across five selected watersheds. The majority of the LULC area changed in watersheds 3479000, 8178700, and 8178050 from 2008 to 2021, while partial changes were observed in watersheds 1115170 and 5527800. The average CV in watersheds was ranked as follows: 3479000 > 8178700 > 8178050 > 1115170 > 5527800. However, the maximum CV in watersheds was ranked as follows: 3479000 (55.84%) > 1115170 (48.56%) > 5527800 (42.12%) > 8178050 (25.65%) > 8178700 (25.26%). The maximum CV indicates the extreme values over the 14 years from 2008 to 2021, while the average CV denotes the typical representation for each watershed. The CV ranges of each watershed are primarily in the range of 0–10, however, the larger CV also occurred due to the significant LUCC.

The average ΔCN across watersheds was ordered as follows: 3479000 > 8178700 > 8178050 > 5527800 > 1115170. The maximum ΔCN for watersheds 1115170 and 3479000 were greater than others caused by high CN areas (open water, CN = 100 in all HSGs) changing to low CN areas (woodlands, CN = 30 in HSG A), and low CN areas (woodlands) converted into high CN areas (high intensity developed areas, CN = 92 in HSG A). The average ΔCN on watershed 5527800 was reduced, while other watersheds were increased. Due to the multiple hydrologic soil-cover complex mapping to a single CN value (i.e., woody wetland and fairly good wood with HSG D have the same CN value), some LULC changed from 2009 to 2020, but CN value remained unchanged.

### CN methods in forest-dominated watersheds

**Fig. 8:**
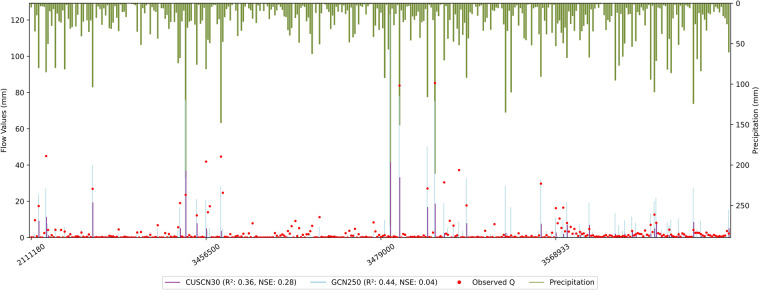
Comparison of the estimated surface flow using curve numbers from CUSCN30 and GCN250 data with observed surface flow based on randomly selected precipitation events.

The CUSCN30 CN values in watersheds 2111180, 3456500, 3479000, and 3568933 were underestimated against observed CN values, raising doubts on the effectiveness of CN values in forest-dominated watersheds (Section 3.1). For further validation, we selected estimated Q from CUSCN30, GCN250, and observed Q using a randomly chosen dataset consisting of 94 P events for each station (Fig. 8). Overall, the estimated Q from CUSCN30 was lower than the observed values, particularly in peak observed Q areas. Conversely, estimated Q of GCN250 adequately captures the peak observed Q but tends to overestimate Q in some cases. In terms of R^2^, estimated Q of CUSCN30 showed a decrease from 0.44 (with an F-statistic of 288.58) to 0.36 (with an F-statistic of 209.90) compared to GCN250. This suggests that GCN250 outperforms CUSCN30 in terms of R². However, the NSE decreased from 0.28 to 0.04 when using GCN250, indicating that, in terms of NSE, GCN250 is less effective than CUSCN30.

The performance disparities between CUSCN30 and GCN250 can be attributed to the source HSG dataset. Most of the wood areas of CUSCN30 are classified as HSGs A and B (CN = 33 and CN = 58, respectively). However, these same areas are categorized as HSGs C and D (CN = 72 and CN = 78, respectively) in GCN250.

In summary, regardless of whether CUSCN30 or GCN250 is used, achieving satisfactory results in estimating Q using the CN method in forest-dominated areas presents notable challenges, as indicated by lower R² and NSE values. Within the CUSCN30 dataset, Lower CN values generally perform well in most events but struggle to fit the variation in Q. Conversely, in the GCN250 dataset the higher CN values are effective in simulating extreme Q events but tend to overestimate in most events. Thus, GCN250 can yield better simulation results in forest-dominated watersheds with higher runoff. Otherwise, CUSCN30 may be more appropriate. However, we do not suggest directly applying the CN method combined with the CUSCN30 dataset for estimating Q in forest-dominated watersheds. This stance is supported by findings from previous research^40,58,59^.

### Limitations and uncertainties

The development of CUSCN30 relied on various datasets, with the accuracy of input data being a key factor influencing the error margin. The accuracy of the NLCD database is over 82%^62,66^, and the CDL dataset is over 87%^67^. However, the accuracies of other datasets, such as SSURGO, HYSOGs250, and NFTD, were not explicitly documented. SSURGO was reported as a superior soil dataset^68–70^, expected to be more accurate than HYSOGs250m in CONUS. Moreover, NFTD provided by the USDA Forest Service has been widely utilized as input data in many studies^71,72^.

Regarding input LULC datasets, disparities exist in their resolution and satellite sources. The CDL and NLCD, derived from Landsat satellite^62,63^, provide a resolution of 30 meters. In contrast, the NFTD based on the MODIS data, has a resolution of 250 meters^73^. Although the Landsat and MODIS images have been fused in many studies and show good performance^74,75^, the distinct data sources inevitably introduce errors due to variations in sensors and spatial resolutions^74,75^. As for the HSG datasets, it’s worth noting that SSURGO’s data collection only covers 91.47% of the CONUS. HYSOGs250m, which represented a great distinction from SSURGO, was employed to fill the gap. Similarly to LULC datasets, The merged HSG data face limitations due to inconsistency between the two data sources.

## Supplementary information

Supplementary information

## Data Availability

The code is publicly available at https://github.com/QiongWuChina/CUSCN30Python.
