# Author Correction: High-resolution Annual Dynamic dataset of Curve Number from 2008 to 2021 over Conterminous United States

**DOI:** 10.1038/s41597-024-03988-5

**Published:** 2024-10-14

**Authors:** Qiong Wu, Jia Yang, Cunxiong Ji, Shanmin Fang

**Affiliations:** 1 Shaanxi Key Laboratory of Earth Surface System and Environmental Carrying Capacity, College of Urban and Environmental Sciences, Northwest University, Xi’an, 710127 China; 2 Institute of Qinling Mountains, Northwest University, Xi’an, 710127 China; 3 Department of Natural Resource Ecology and Management, Oklahoma State University, Stillwater, OK 74074 USA; 4 Shaanxi Water Environment Design Group Co., Ltd., Xi’an, 710021 China

**Keywords:** Hydrology, Hydrology

Correction to: *Scientific Data* 10.1038/s41597-024-03044-2, published online 15 February 2024

John Ramirez Avila, who conceptualized the study and contributed to an earlier draft of this manuscript, was omitted from the original author list and has been added to the pdf and HTML versions of the paper. An explanation of John Ramirez Avila’s role has also been added to the Author Contributions statement. This statement in the Acknowledgements section, which incorrectly described his role, has been removed:

“Additionally, the dataset presented in this study was completed and released during the tenure at Mississippi State University, and the authors would like to express gratitude to Dr. Ramirez for data upload and data storage.”

And replaced with this note:

This study was conceptualized to contribute to the perspective and goals of the Curve Number Hydrology Task Committee, which is organized under the Watershed Management Technical Committee within the ASCE Environmental and Water Resources Institute (EWRI).

The original article has been corrected.

