# Supplementary material for: High-resolution Annual Dynamic dataset of Curve Number from 2008 to 2021 over Conterminous United States
Source: Sci Data. 2024 Feb 15;11:207. doi: 10.1038/s41597-024-03044-2 (PMC10869687; doi:10.1038/s41597-024-03044-2)
Supplement: Supplementary file 1 — Supplementary information [file 41597_2024_3044_MOESM1_ESM.docx]

### Supplementary information

Contents

1 Supplementary Table 1. Detailed information on the 10 watersheds for validation purposes.

2 Supplementary Table 2. Observed CN calculated by four simulated surface flow methods and its mean value with an initial abstraction coefficient of 0.2.

3 Supplementary Table 3. Detailed information of randomly selected validation data for the 10 watersheds.

4 Supplementary Table 4. Detailed information of randomly selected validation data for forest-dominated watersheds.

**Supplementary Table 1.** Detailed information on the 10 watersheds for validation purposes.

| Watershed | Station Name | State | Available Time | Latitude | Longitude | Mainly Types of LULC (2021) | HSG | Slope (range, mean and SD) |
| --- | --- | --- | --- | --- | --- | --- | --- | --- |
| 1115170 | Moswansicut Stream Nr North Scituate | RI | 2020-2021 | 41.84 | -71.58 | Fairly good wood (35.3%), open water (13.2%), developed low intensity (11.3%) | B (43.8%), D(31%), C (17.1%), D (8.0%) | 0.14-0.30, 0.14, 1.07 |
| 2111180 | Elk Creek At Elkville | NC | 2008-2021 | 36.07 | -81.40 | Fairly good wood (90.1%) | B (73.6%), C (15.7%), A (9.9%), D (0.8%) | 0.14-1.22, 0.27, 12.03 |
| 3456500 | East Fork Pigeon River Near Canton | NC | 2008-2022 | 35.46 | -82.87 | Fairly good wood (88.9%) | B (68.1%), A (28.9%), A (2.5%), D (0.5%) | 0.14-1.26, 0.34, 13.85 |
| 3479000 | Watauga River Near Sugar Grove | NC | 2016-2021 | 36.24 | -81.82 | Fairly good wood (72.3%), developed open space (12.8%) | B (64.1%), A (32.8%), D (2.0%), D (1.1%) | 0.14-0.96, 0.26, 11.28 |
| 3568933 | Lookout Creek Near New England | GA | 2020-2021 | 34.90 | -85.46 | Fairly good wood (66.9%), pasture (12.8%) | B (42.3%), D (29.0%), A (20.9%), C (7.8%) | 0.14-1.40, 0.19, 8.86 |
| 5527800 | Des Plaines River At Russell | IL | 2008-2022 | 42.49 | -87.93 | Fair CBR (22.0%), fair row crops (17.2%), pasture (10.7%), fair wood (10.5%) | C (51.2%), D (46.8%), B (1.8%), A (0.1%) | 0.14-0.36, 0.14, 0.36 |
| 5590050 | Copper Slough At Champaign | IL | 2008-2023 | 40.10 | -88.31 | Developed low intensity (32.5%), developed medium intensity (29.2%), fair CBR (13.6%), developed high intensity (11.7%) | D (61.7%), C (38.3%) | 0.14-0.16, 0.14, 0.02 |
| 8178050 | San Antonio Rv At Mitchell St, San Antonio | TX | 2010-2021 | 29.39 | -98.49 | Developed medium intensity (27.9%), developed low intensity (26.8%), developed high intensity (21.5%), developed open space (15.8%) | D (91.2%), B (6.5%),C (2.3%) | 0.14-0.90, 0.14, 1.39 |
| 8178700 | Salado Ck At Loop 410, San Antonio | TX | 2011-2021 | 29.52 | -98.43 | Developed medium intensity (22.0%), fairly good wood (18.6%), developed low intensity (17.6%), developed open space (13.2%), brush mixture (11.4%) | D (87.5%), C (6.8%), B (5.7%) | 0.14-0.50, 0.14, 1.05 |
| 9419740 | C-1 Channel Nr Warm Spgs Rd At Henderson | NV | 2008-2021 | 36.04 | -114.96 | Brush mixture (68.9%), developed medium intensity (14.2%) | D (54.2%), A (45.1%) | 0.14-1.4, 0.18, 8.52 |

**Supplementary Table 2.** Observed CN calculated by four simulated surface flow methods and its mean value with an initial abstraction coefficient of 0.2.

| No. | Station | Area (sq mi) | observed CN | | | | |
| --- | --- | --- | --- | --- | --- | --- | --- |
|  |  |  | PART | HySEP-Fixed | HySEP-LocMin | HySEP-Slide | Mean |
| 1 | 1115170 | 3.25 | 87.04 | 87.17 | 85.72 | 85.72 | 86.41 |
| 2 | 2111180 | 50.90 | 83.72 | 84.24 | 84.58 | 84.45 | 84.25 |
| 3 | 3456500 | 51.50 | 86.75 | 87.63 | 88.09 | 87.85 | 87.58 |
| 4 | 3479000 | 92.10 | 82.23 | 83.12 | 83.52 | 83.33 | 83.05 |
| 5 | 3568933 | 149.00 | 81.47 | 81.63 | 81.40 | 81.73 | 81.56 |
| 6 | 5527800 | 123.00 | 85.22 | 85.50 | 86.24 | 85.70 | 85.66 |
| 7 | 5590050 | 7.98 | 91.15 | 90.78 | 91.39 | 90.91 | 91.06 |
| 8 | 8178050 | 42.40 | 89.82 | 89.84 | 89.91 | 89.85 | 89.86 |
| 9 | 8178700 | 137.00 | 87.20 | 87.16 | 87.20 | 87.18 | 87.19 |
| 10 | 9419740 | 3.78 | 94.67 | 94.67 | 94.66 | 94.67 | 94.67 |

**Supplementary Table 3.** Detailed information of randomly selected validation data for the 10 watersheds.

| Station | Q mm | P mm | Q CUSCN30 I | Q CUSCN30 II | Q CUSCN30 III | Q GCN-ARCI | Q GCN-ARCII | Q GCN-ARCIII | Q CUSCN30 | Q GCN |
| --- | --- | --- | --- | --- | --- | --- | --- | --- | --- | --- |
| 1115170 | 19.57 | 21.59 | 0.00 | 0.01 | 2.96 | 0.00 | 0.00 | 0.94 | 0.01 | 0.00 |
| 1115170 | 6.10 | 7.37 | 0.00 | 0.00 | 0.00 | 0.00 | 0.00 | 0.00 | 0.00 | 0.00 |
| 1115170 | 5.75 | 7.62 | 0.00 | 0.00 | 0.00 | 0.00 | 0.00 | 0.00 | 0.00 | 0.00 |
| 1115170 | 4.43 | 6.35 | 0.00 | 0.00 | 0.00 | 0.00 | 0.00 | 0.00 | 0.00 | 0.00 |
| 1115170 | 3.65 | 18.03 | 0.00 | 0.00 | 1.66 | 0.00 | 0.00 | 0.32 | 0.00 | 0.00 |
| 1115170 | 50.35 | 82.04 | 4.79 | 22.82 | 46.10 | 0.61 | 13.02 | 35.07 | 22.82 | 13.02 |
| 1115170 | 21.38 | 69.85 | 2.18 | 15.82 | 35.79 | 0.02 | 8.07 | 26.10 | 15.82 | 8.07 |
| 1115170 | 19.63 | 22.86 | 0.00 | 0.04 | 3.49 | 0.00 | 0.00 | 1.22 | 0.04 | 0.00 |
| 1115170 | 3.54 | 49.28 | 0.04 | 6.18 | 19.62 | 0.00 | 2.06 | 12.71 | 6.18 | 2.06 |
| 1115170 | 7.13 | 15.49 | 0.00 | 0.00 | 0.93 | 0.00 | 0.00 | 0.07 | 0.00 | 0.00 |
| 1115170 | 6.80 | 59.69 | 0.74 | 10.66 | 27.57 | 0.00 | 4.69 | 19.16 | 0.74 | 0.00 |
| 1115170 | 13.70 | 69.85 | 2.18 | 15.82 | 35.79 | 0.02 | 8.07 | 26.10 | 15.82 | 8.07 |
| 1115170 | 3.48 | 10.92 | 0.00 | 0.00 | 0.11 | 0.00 | 0.00 | 0.00 | 0.00 | 0.00 |
| 1115170 | 3.61 | 4.57 | 0.00 | 0.00 | 0.00 | 0.00 | 0.00 | 0.00 | 0.00 | 0.00 |
| 1115170 | 4.97 | 76.20 | 3.43 | 19.36 | 41.11 | 0.22 | 10.53 | 30.70 | 3.43 | 0.22 |
| 1115170 | 13.21 | 15.24 | 0.00 | 0.00 | 1.00 | 0.00 | 0.00 | 0.06 | 0.00 | 0.00 |
| 1115170 | 17.86 | 55.88 | 0.55 | 9.59 | 25.44 | 0.00 | 3.62 | 16.71 | 9.59 | 3.62 |
| 1115170 | 16.83 | 38.10 | 0.00 | 2.83 | 12.47 | 0.00 | 0.36 | 6.79 | 2.83 | 0.36 |
| 1115170 | 8.54 | 18.03 | 0.00 | 0.00 | 1.85 | 0.00 | 0.00 | 0.32 | 0.00 | 0.00 |
| 1115170 | 15.78 | 30.48 | 0.00 | 1.03 | 7.74 | 0.00 | 0.00 | 3.56 | 1.03 | 0.00 |
| 1115170 | 12.92 | 13.46 | 0.00 | 0.00 | 0.57 | 0.00 | 0.00 | 0.00 | 0.57 | 0.00 |
| 1115170 | 11.75 | 104.14 | 12.60 | 38.70 | 66.91 | 3.61 | 23.97 | 52.61 | 12.60 | 3.61 |
| 1115170 | 41.12 | 52.32 | 0.27 | 8.00 | 22.68 | 0.00 | 2.73 | 14.51 | 8.00 | 2.73 |
| 2111180 | 0.51 | 43.43 | 0.00 | 0.37 | 7.41 | 0.00 | 4.95 | 16.75 | 0.00 | 0.00 |
| 2111180 | 5.86 | 73.66 | 0.00 | 7.04 | 25.18 | 3.68 | 19.86 | 40.97 | 0.00 | 3.68 |
| 2111180 | 0.05 | 2.54 | 0.00 | 0.00 | 0.00 | 0.00 | 0.00 | 0.00 | 0.00 | 0.00 |
| 2111180 | 0.00 | 0.51 | 0.00 | 0.00 | 0.00 | 0.00 | 0.00 | 0.00 | 0.00 | 0.00 |
| 2111180 | 0.51 | 10.67 | 0.00 | 0.00 | 0.00 | 0.00 | 0.00 | 0.18 | 0.00 | 0.00 |
| 2111180 | 0.08 | 6.10 | 0.00 | 0.00 | 0.00 | 0.00 | 0.00 | 0.00 | 0.00 | 0.00 |
| 2111180 | 0.66 | 17.27 | 0.00 | 0.00 | 0.05 | 0.00 | 0.00 | 1.78 | 0.00 | 0.00 |
| 2111180 | 4.21 | 62.74 | 0.00 | 3.77 | 17.98 | 1.57 | 13.70 | 31.77 | 0.00 | 1.57 |
| 2111180 | 1.05 | 32.00 | 0.00 | 0.00 | 2.93 | 0.00 | 1.54 | 9.08 | 0.00 | 1.54 |
| 2111180 | 0.13 | 8.38 | 0.00 | 0.00 | 0.00 | 0.00 | 0.00 | 0.00 | 0.00 | 0.00 |
| 2111180 | 0.93 | 30.99 | 0.00 | 0.00 | 2.67 | 0.00 | 1.32 | 8.46 | 0.00 | 1.32 |
| 2111180 | 0.31 | 10.67 | 0.00 | 0.00 | 0.00 | 0.00 | 0.00 | 0.18 | 0.00 | 0.00 |
| 2111180 | 4.47 | 34.54 | 0.00 | 0.00 | 3.79 | 0.00 | 2.15 | 10.67 | 0.00 | 2.15 |
| 2111180 | 0.00 | 0.25 | 0.00 | 0.00 | 0.00 | 0.00 | 0.00 | 0.00 | 0.00 | 0.00 |
| 2111180 | 0.28 | 16.26 | 0.00 | 0.00 | 0.01 | 0.00 | 0.00 | 1.44 | 0.00 | 0.00 |
| 2111180 | 0.22 | 15.49 | 0.00 | 0.00 | 0.00 | 0.00 | 0.00 | 1.21 | 0.00 | 0.00 |
| 2111180 | 6.28 | 80.26 | 0.02 | 9.19 | 29.51 | 5.32 | 23.90 | 46.69 | 0.02 | 5.32 |
| 2111180 | 0.12 | 5.08 | 0.00 | 0.00 | 0.00 | 0.00 | 0.00 | 0.00 | 0.00 | 0.00 |
| 2111180 | 0.00 | 2.79 | 0.00 | 0.00 | 0.00 | 0.00 | 0.00 | 0.00 | 0.00 | 0.00 |
| 2111180 | 0.35 | 30.23 | 0.00 | 0.00 | 2.36 | 0.00 | 1.16 | 8.01 | 0.00 | 1.16 |
| 2111180 | 0.00 | 0.25 | 0.00 | 0.00 | 0.00 | 0.00 | 0.00 | 0.00 | 0.00 | 0.00 |
| 2111180 | 0.01 | 0.51 | 0.00 | 0.00 | 0.00 | 0.00 | 0.00 | 0.00 | 0.00 | 0.00 |
| 2111180 | 19.01 | 67.82 | 0.00 | 5.22 | 21.31 | 2.45 | 16.47 | 36.00 | 5.22 | 16.47 |
| 3456500 | 1.08 | 4.57 | 0.00 | 0.00 | 0.00 | 0.00 | 0.00 | 0.00 | 0.00 | 0.00 |
| 3456500 | 1.04 | 21.08 | 0.00 | 0.00 | 0.00 | 0.00 | 0.00 | 2.32 | 0.00 | 0.00 |
| 3456500 | 0.02 | 7.87 | 0.00 | 0.00 | 0.00 | 0.00 | 0.00 | 0.00 | 0.00 | 0.00 |
| 3456500 | 0.90 | 15.49 | 0.00 | 0.00 | 0.00 | 0.00 | 0.00 | 0.70 | 0.00 | 0.00 |
| 3456500 | 0.22 | 8.13 | 0.00 | 0.00 | 0.00 | 0.00 | 0.00 | 0.00 | 0.00 | 0.00 |
| 3456500 | 0.05 | 0.76 | 0.00 | 0.00 | 0.00 | 0.00 | 0.00 | 0.00 | 0.00 | 0.00 |
| 3456500 | 4.84 | 83.06 | 0.00 | 5.29 | 23.65 | 4.05 | 21.69 | 44.96 | 0.00 | 4.05 |
| 3456500 | 10.84 | 47.75 | 0.00 | 0.02 | 5.74 | 0.00 | 4.84 | 17.23 | 0.02 | 4.84 |
| 3456500 | 22.08 | 111.76 | 0.15 | 15.03 | 43.08 | 12.59 | 40.19 | 70.14 | 0.15 | 12.59 |
| 3456500 | 0.00 | 4.83 | 0.00 | 0.00 | 0.00 | 0.00 | 0.00 | 0.00 | 0.00 | 0.00 |
| 3456500 | 1.01 | 27.18 | 0.00 | 0.00 | 0.44 | 0.00 | 0.22 | 4.88 | 0.00 | 0.22 |
| 3456500 | 0.04 | 1.52 | 0.00 | 0.00 | 0.00 | 0.00 | 0.00 | 0.00 | 0.00 | 0.00 |
| 3456500 | 0.00 | 1.27 | 0.00 | 0.00 | 0.00 | 0.00 | 0.00 | 0.00 | 0.00 | 0.00 |
| 3456500 | 0.21 | 6.86 | 0.00 | 0.00 | 0.00 | 0.00 | 0.00 | 0.00 | 0.00 | 0.00 |
| 3456500 | 0.10 | 4.32 | 0.00 | 0.00 | 0.00 | 0.00 | 0.00 | 0.00 | 0.00 | 0.00 |
| 3456500 | 0.00 | 1.27 | 0.00 | 0.00 | 0.00 | 0.00 | 0.00 | 0.00 | 0.00 | 0.00 |
| 3456500 | 0.01 | 2.03 | 0.00 | 0.00 | 0.00 | 0.00 | 0.00 | 0.00 | 0.00 | 0.00 |
| 3456500 | 0.00 | 0.25 | 0.00 | 0.00 | 0.00 | 0.00 | 0.00 | 0.00 | 0.00 | 0.00 |
| 3456500 | 2.44 | 14.99 | 0.00 | 0.00 | 0.00 | 0.00 | 0.00 | 0.59 | 0.00 | 0.00 |
| 3456500 | 0.00 | 3.56 | 0.00 | 0.00 | 0.00 | 0.00 | 0.00 | 0.00 | 0.00 | 0.00 |
| 3456500 | 0.00 | 2.03 | 0.00 | 0.00 | 0.00 | 0.00 | 0.00 | 0.00 | 0.00 | 0.00 |
| 3456500 | 1.34 | 23.62 | 0.00 | 0.00 | 0.10 | 0.00 | 0.02 | 3.30 | 0.00 | 0.02 |
| 3456500 | 1.33 | 27.43 | 0.00 | 0.00 | 0.47 | 0.00 | 0.25 | 5.00 | 0.00 | 0.25 |
| 3479000 | 1.84 | 11.94 | 0.00 | 0.00 | 0.00 | 0.00 | 0.00 | 0.43 | 0.00 | 0.00 |
| 3479000 | 0.00 | 0.25 | 0.00 | 0.00 | 0.00 | 0.00 | 0.00 | 0.00 | 0.00 | 0.00 |
| 3479000 | 0.16 | 42.42 | 0.00 | 0.00 | 3.65 | 0.00 | 4.94 | 16.57 | 0.00 | 0.00 |
| 3479000 | 3.78 | 40.39 | 0.00 | 0.00 | 3.21 | 0.00 | 4.21 | 15.11 | 0.00 | 4.21 |
| 3479000 | 0.62 | 13.72 | 0.00 | 0.00 | 0.00 | 0.00 | 0.00 | 0.83 | 0.00 | 0.00 |
| 3479000 | 1.14 | 16.00 | 0.00 | 0.00 | 0.00 | 0.00 | 0.00 | 1.50 | 0.00 | 0.00 |
| 3479000 | 0.86 | 14.48 | 0.00 | 0.00 | 0.00 | 0.00 | 0.00 | 1.04 | 0.00 | 0.00 |
| 3479000 | 0.00 | 1.52 | 0.00 | 0.00 | 0.00 | 0.00 | 0.00 | 0.00 | 0.00 | 0.00 |
| 3479000 | 6.17 | 14.22 | 0.00 | 0.00 | 0.00 | 0.00 | 0.00 | 0.97 | 0.00 | 0.00 |
| 3479000 | 0.00 | 23.88 | 0.00 | 0.00 | 0.16 | 0.00 | 0.30 | 4.84 | 0.00 | 0.30 |
| 3479000 | 0.60 | 17.02 | 0.00 | 0.00 | 0.00 | 0.00 | 0.00 | 1.85 | 0.00 | 0.00 |
| 3479000 | 0.65 | 12.70 | 0.00 | 0.00 | 0.00 | 0.00 | 0.00 | 0.59 | 0.00 | 0.00 |
| 3479000 | 0.14 | 27.18 | 0.00 | 0.00 | 0.36 | 0.00 | 0.75 | 6.61 | 0.00 | 0.75 |
| 3479000 | 1.15 | 38.35 | 0.00 | 0.00 | 2.91 | 0.00 | 3.53 | 13.68 | 0.00 | 3.53 |
| 3479000 | 1.30 | 24.13 | 0.00 | 0.00 | 0.12 | 0.00 | 0.33 | 4.97 | 0.00 | 0.33 |
| 3479000 | 0.22 | 7.62 | 0.00 | 0.00 | 0.00 | 0.00 | 0.00 | 0.00 | 0.00 | 0.00 |
| 3479000 | 0.49 | 2.29 | 0.00 | 0.00 | 0.00 | 0.00 | 0.00 | 0.00 | 0.00 | 0.00 |
| 3479000 | 0.00 | 6.35 | 0.00 | 0.00 | 0.00 | 0.00 | 0.00 | 0.00 | 0.00 | 0.00 |
| 3479000 | 0.01 | 1.02 | 0.00 | 0.00 | 0.00 | 0.00 | 0.00 | 0.00 | 0.00 | 0.00 |
| 3479000 | 0.37 | 20.83 | 0.00 | 0.00 | 0.01 | 0.00 | 0.06 | 3.38 | 0.00 | 0.06 |
| 3479000 | 1.84 | 2.79 | 0.00 | 0.00 | 0.00 | 0.00 | 0.00 | 0.00 | 0.00 | 0.00 |
| 3479000 | 0.00 | 1.02 | 0.00 | 0.00 | 0.00 | 0.00 | 0.00 | 0.00 | 0.00 | 0.00 |
| 3479000 | 0.22 | 13.72 | 0.00 | 0.00 | 0.00 | 0.00 | 0.00 | 0.83 | 0.00 | 0.00 |
| 3568933 | 3.58 | 25.91 | 0.00 | 0.00 | 2.08 | 0.00 | 0.96 | 6.90 | 0.00 | 0.96 |
| 3568933 | 4.15 | 124.46 | 8.59 | 36.16 | 70.05 | 27.38 | 61.12 | 91.35 | 8.59 | 27.38 |
| 3568933 | 0.99 | 7.62 | 0.00 | 0.00 | 0.00 | 0.00 | 0.00 | 0.02 | 0.00 | 0.00 |
| 3568933 | 1.47 | 2.54 | 0.00 | 0.00 | 0.00 | 0.00 | 0.00 | 0.00 | 0.00 | 0.00 |
| 3568933 | 1.28 | 25.91 | 0.00 | 0.00 | 1.97 | 0.00 | 0.96 | 6.90 | 0.00 | 0.96 |
| 3568933 | 0.84 | 66.04 | 0.00 | 6.54 | 23.15 | 3.71 | 18.55 | 37.69 | 0.00 | 3.71 |
| 3568933 | 4.33 | 44.20 | 0.00 | 1.18 | 9.99 | 0.22 | 6.94 | 19.62 | 1.18 | 6.94 |
| 3568933 | 1.90 | 84.07 | 0.88 | 14.13 | 36.86 | 9.17 | 30.38 | 53.72 | 0.88 | 9.17 |
| 3568933 | 0.32 | 4.06 | 0.00 | 0.00 | 0.00 | 0.00 | 0.00 | 0.00 | 0.00 | 0.00 |
| 3568933 | 1.90 | 5.08 | 0.00 | 0.00 | 0.00 | 0.00 | 0.00 | 0.00 | 0.00 | 0.00 |
| 3568933 | 0.92 | 4.57 | 0.00 | 0.00 | 0.00 | 0.00 | 0.00 | 0.00 | 0.00 | 0.00 |
| 3568933 | 1.19 | 34.80 | 0.00 | 0.10 | 5.18 | 0.00 | 3.31 | 12.67 | 0.10 | 3.31 |
| 3568933 | 1.15 | 1.52 | 0.00 | 0.00 | 0.00 | 0.00 | 0.00 | 0.00 | 0.00 | 0.00 |
| 3568933 | 16.52 | 49.53 | 0.00 | 2.02 | 12.65 | 0.70 | 9.42 | 23.84 | 2.02 | 9.42 |
| 3568933 | 0.54 | 1.27 | 0.00 | 0.00 | 0.00 | 0.00 | 0.00 | 0.00 | 0.00 | 0.00 |
| 3568933 | 0.26 | 0.51 | 0.00 | 0.00 | 0.00 | 0.00 | 0.00 | 0.00 | 0.00 | 0.00 |
| 3568933 | 1.13 | 13.97 | 0.00 | 0.00 | 0.01 | 0.00 | 0.00 | 1.26 | 0.00 | 0.00 |
| 3568933 | 0.43 | 6.60 | 0.00 | 0.00 | 0.00 | 0.00 | 0.00 | 0.00 | 0.00 | 0.00 |
| 3568933 | 3.43 | 29.97 | 0.00 | 0.00 | 3.28 | 0.00 | 1.88 | 9.42 | 0.00 | 1.88 |
| 3568933 | 0.67 | 2.54 | 0.00 | 0.00 | 0.00 | 0.00 | 0.00 | 0.00 | 0.00 | 0.00 |
| 3568933 | 1.95 | 6.35 | 0.00 | 0.00 | 0.00 | 0.00 | 0.00 | 0.00 | 0.00 | 0.00 |
| 3568933 | 1.48 | 12.19 | 0.00 | 0.00 | 0.00 | 0.00 | 0.00 | 0.74 | 0.00 | 0.00 |
| 3568933 | 3.61 | 53.85 | 0.00 | 2.99 | 15.22 | 1.28 | 11.61 | 27.35 | 2.99 | 11.61 |
| 5527800 | 1.07 | 39.37 | 1.55 | 10.51 | 22.35 | 0.07 | 5.51 | 16.85 | 10.51 | 5.51 |
| 5527800 | 0.87 | 6.86 | 0.00 | 0.00 | 0.40 | 0.00 | 0.00 | 0.01 | 0.00 | 0.00 |
| 5527800 | 0.16 | 29.97 | 0.22 | 5.51 | 14.54 | 0.00 | 2.21 | 10.09 | 5.51 | 2.21 |
| 5527800 | 0.10 | 2.54 | 0.00 | 0.00 | 0.00 | 0.00 | 0.00 | 0.00 | 0.00 | 0.00 |
| 5527800 | 0.00 | 0.51 | 0.00 | 0.00 | 0.00 | 0.00 | 0.00 | 0.00 | 0.00 | 0.00 |
| 5527800 | 1.24 | 31.50 | 0.36 | 6.25 | 15.77 | 0.00 | 2.66 | 11.13 | 6.25 | 2.66 |
| 5527800 | 0.00 | 1.52 | 0.00 | 0.00 | 0.00 | 0.00 | 0.00 | 0.00 | 0.00 | 0.00 |
| 5527800 | 0.00 | 5.08 | 0.00 | 0.00 | 0.05 | 0.00 | 0.00 | 0.00 | 0.00 | 0.00 |
| 5527800 | 0.01 | 11.68 | 0.00 | 0.05 | 2.16 | 0.00 | 0.00 | 0.77 | 0.05 | 0.00 |
| 5527800 | 0.55 | 8.64 | 0.00 | 0.00 | 0.83 | 0.00 | 0.00 | 0.15 | 0.00 | 0.00 |
| 5527800 | 1.34 | 1.78 | 0.00 | 0.00 | 0.00 | 0.00 | 0.00 | 0.00 | 0.00 | 0.00 |
| 5527800 | 0.35 | 0.76 | 0.00 | 0.00 | 0.00 | 0.00 | 0.00 | 0.00 | 0.00 | 0.00 |
| 5527800 | 0.00 | 2.03 | 0.00 | 0.00 | 0.00 | 0.00 | 0.00 | 0.00 | 0.00 | 0.00 |
| 5527800 | 0.09 | 33.27 | 0.54 | 7.09 | 17.16 | 0.00 | 3.23 | 12.37 | 0.54 | 0.00 |
| 5527800 | 0.00 | 9.65 | 0.00 | 0.00 | 1.21 | 0.00 | 0.00 | 0.31 | 0.00 | 0.00 |
| 5527800 | 0.00 | 19.81 | 0.00 | 1.50 | 6.86 | 0.00 | 0.21 | 4.02 | 1.50 | 0.21 |
| 5527800 | 0.08 | 18.03 | 0.00 | 1.01 | 5.65 | 0.00 | 0.07 | 3.15 | 1.01 | 0.07 |
| 5527800 | 0.01 | 0.25 | 0.00 | 0.00 | 0.00 | 0.00 | 0.00 | 0.00 | 0.00 | 0.00 |
| 5527800 | 1.23 | 49.78 | 4.24 | 17.20 | 31.63 | 1.03 | 10.35 | 25.11 | 4.24 | 1.03 |
| 5527800 | 1.99 | 86.87 | 20.73 | 45.79 | 66.38 | 11.38 | 33.90 | 57.77 | 20.73 | 11.38 |
| 5527800 | 0.00 | 5.59 | 0.00 | 0.00 | 0.10 | 0.00 | 0.00 | 0.00 | 0.00 | 0.00 |
| 5527800 | 0.01 | 10.67 | 0.00 | 0.01 | 1.65 | 0.00 | 0.00 | 0.52 | 0.01 | 0.00 |
| 5527800 | 0.06 | 0.25 | 0.00 | 0.00 | 0.00 | 0.00 | 0.00 | 0.00 | 0.00 | 0.00 |
| 5590050 | 0.04 | 0.25 | 0.00 | 0.00 | 0.00 | 0.00 | 0.00 | 0.00 | 0.00 | 0.00 |
| 5590050 | 2.64 | 18.29 | 0.00 | 2.45 | 7.93 | 0.00 | 0.89 | 5.37 | 2.45 | 0.89 |
| 5590050 | 4.20 | 57.91 | 11.93 | 29.23 | 43.68 | 6.96 | 21.81 | 37.90 | 11.93 | 6.96 |
| 5590050 | 0.15 | 1.02 | 0.00 | 0.00 | 0.00 | 0.00 | 0.00 | 0.00 | 0.00 | 0.00 |
| 5590050 | 1.24 | 21.34 | 0.10 | 3.84 | 10.40 | 0.00 | 1.69 | 7.36 | 3.84 | 1.69 |
| 5590050 | 0.28 | 8.89 | 0.00 | 0.05 | 1.75 | 0.00 | 0.00 | 0.76 | 0.05 | 0.00 |
| 5590050 | 0.24 | 5.33 | 0.00 | 0.00 | 0.31 | 0.00 | 0.00 | 0.04 | 0.00 | 0.00 |
| 5590050 | 1.29 | 2.79 | 0.00 | 0.00 | 0.00 | 0.00 | 0.00 | 0.00 | 0.00 | 0.00 |
| 5590050 | 18.55 | 57.66 | 11.80 | 29.02 | 43.43 | 6.87 | 21.63 | 37.67 | 11.80 | 6.87 |
| 5590050 | 3.93 | 26.92 | 0.59 | 6.39 | 14.68 | 0.03 | 3.65 | 11.38 | 6.39 | 3.65 |
| 5590050 | 12.20 | 30.48 | 1.22 | 8.46 | 17.77 | 0.25 | 5.19 | 14.13 | 8.46 | 5.19 |
| 5590050 | 0.10 | 0.25 | 0.00 | 0.00 | 0.00 | 0.00 | 0.00 | 0.00 | 0.00 | 0.00 |
| 5590050 | 13.72 | 49.53 | 7.29 | 21.67 | 35.09 | 4.10 | 16.05 | 30.32 | 21.67 | 16.05 |
| 5590050 | 0.00 | 0.51 | 0.00 | 0.00 | 0.00 | 0.00 | 0.00 | 0.00 | 0.00 | 0.00 |
| 5590050 | 4.95 | 25.40 | 0.38 | 5.52 | 13.35 | 0.00 | 3.06 | 10.25 | 5.52 | 3.06 |
| 5590050 | 0.07 | 0.51 | 0.00 | 0.00 | 0.00 | 0.00 | 0.00 | 0.00 | 0.00 | 0.00 |
| 5590050 | 0.47 | 14.22 | 0.00 | 1.07 | 5.02 | 0.00 | 0.19 | 3.03 | 1.07 | 0.19 |
| 5590050 | 0.07 | 0.51 | 0.00 | 0.00 | 0.00 | 0.00 | 0.00 | 0.00 | 0.00 | 0.00 |
| 5590050 | 1.15 | 7.37 | 0.00 | 0.00 | 1.05 | 0.00 | 0.00 | 0.35 | 0.00 | 0.00 |
| 5590050 | 0.91 | 10.16 | 0.00 | 0.16 | 2.35 | 0.00 | 0.00 | 1.19 | 0.16 | 0.00 |
| 5590050 | 6.15 | 41.40 | 4.56 | 16.23 | 28.01 | 1.95 | 10.97 | 23.19 | 4.56 | 1.95 |
| 5590050 | 0.09 | 3.05 | 0.00 | 0.00 | 0.00 | 0.00 | 0.00 | 0.00 | 0.00 | 0.00 |
| 5590050 | 0.11 | 0.76 | 0.00 | 0.00 | 0.00 | 0.00 | 0.00 | 0.00 | 0.00 | 0.00 |
| 8178050 | 0.81 | 4.83 | 0.00 | 0.00 | 0.22 | 0.00 | 0.00 | 0.13 | 0.00 | 0.00 |
| 8178050 | 1.10 | 10.16 | 0.00 | 0.36 | 2.88 | 0.00 | 0.07 | 2.02 | 0.36 | 0.07 |
| 8178050 | 0.11 | 0.51 | 0.00 | 0.00 | 0.00 | 0.00 | 0.00 | 0.00 | 0.00 | 0.00 |
| 8178050 | 0.26 | 4.83 | 0.00 | 0.00 | 0.22 | 0.00 | 0.00 | 0.13 | 0.00 | 0.00 |
| 8178050 | 1.33 | 22.10 | 0.05 | 3.64 | 10.37 | 0.05 | 3.39 | 10.02 | 3.64 | 3.39 |
| 8178050 | 1.12 | 9.91 | 0.00 | 0.16 | 2.31 | 0.00 | 0.05 | 1.89 | 0.16 | 0.05 |
| 8178050 | 7.90 | 65.79 | 15.78 | 35.39 | 50.96 | 14.64 | 33.22 | 49.46 | 15.78 | 14.64 |
| 8178050 | 5.86 | 43.94 | 5.36 | 17.89 | 30.20 | 4.76 | 16.35 | 28.93 | 5.36 | 4.76 |
| 8178050 | 0.02 | 0.51 | 0.00 | 0.00 | 0.00 | 0.00 | 0.00 | 0.00 | 0.00 | 0.00 |
| 8178050 | 0.11 | 5.59 | 0.00 | 0.00 | 0.43 | 0.00 | 0.00 | 0.28 | 0.00 | 0.00 |
| 8178050 | 0.13 | 9.14 | 0.00 | 0.08 | 1.91 | 0.00 | 0.01 | 1.53 | 0.08 | 0.01 |
| 8178050 | 0.02 | 0.76 | 0.00 | 0.00 | 0.00 | 0.00 | 0.00 | 0.00 | 0.00 | 0.00 |
| 8178050 | 1.24 | 11.68 | 0.00 | 0.41 | 3.26 | 0.00 | 0.24 | 2.83 | 0.41 | 0.24 |
| 8178050 | 0.01 | 1.02 | 0.00 | 0.00 | 0.00 | 0.00 | 0.00 | 0.00 | 0.00 | 0.00 |
| 8178050 | 2.94 | 18.03 | 0.00 | 1.98 | 7.23 | 0.00 | 1.81 | 6.96 | 1.98 | 1.81 |
| 8178050 | 52.52 | 146.05 | 70.05 | 106.43 | 128.51 | 70.89 | 105.60 | 127.99 | 70.05 | 70.89 |
| 8178050 | 12.81 | 31.75 | 1.60 | 9.48 | 19.10 | 1.30 | 8.38 | 18.02 | 9.48 | 8.38 |
| 8178050 | 19.94 | 37.08 | 3.00 | 12.96 | 23.87 | 2.59 | 11.69 | 22.72 | 12.96 | 11.69 |
| 8178050 | 0.05 | 0.51 | 0.00 | 0.00 | 0.00 | 0.00 | 0.00 | 0.00 | 0.00 | 0.00 |
| 8178050 | 5.07 | 23.62 | 0.22 | 4.73 | 12.00 | 0.15 | 4.08 | 11.23 | 4.73 | 4.08 |
| 8178050 | 0.11 | 4.06 | 0.00 | 0.00 | 0.05 | 0.00 | 0.00 | 0.03 | 0.00 | 0.00 |
| 8178050 | 0.03 | 0.76 | 0.00 | 0.00 | 0.00 | 0.00 | 0.00 | 0.00 | 0.00 | 0.00 |
| 8178050 | 0.19 | 1.78 | 0.00 | 0.00 | 0.00 | 0.00 | 0.00 | 0.00 | 0.00 | 0.00 |
| 8178700 | 0.00 | 0.25 | 0.00 | 0.00 | 0.00 | 0.00 | 0.00 | 0.00 | 0.00 | 0.00 |
| 8178700 | 0.00 | 0.51 | 0.00 | 0.00 | 0.00 | 0.00 | 0.00 | 0.00 | 0.00 | 0.00 |
| 8178700 | 38.53 | 77.72 | 15.34 | 37.62 | 57.20 | 12.73 | 33.27 | 53.76 | 37.62 | 33.27 |
| 8178700 | 0.04 | 6.10 | 0.00 | 0.00 | 0.23 | 0.00 | 0.00 | 0.04 | 0.00 | 0.00 |
| 8178700 | 6.74 | 88.65 | 22.51 | 48.13 | 68.67 | 17.97 | 41.78 | 64.02 | 22.51 | 17.97 |
| 8178700 | 2.95 | 15.49 | 0.00 | 0.55 | 4.25 | 0.00 | 0.14 | 3.01 | 0.55 | 0.14 |
| 8178700 | 0.00 | 0.25 | 0.00 | 0.00 | 0.00 | 0.00 | 0.00 | 0.00 | 0.00 | 0.00 |
| 8178700 | 21.07 | 70.10 | 12.74 | 32.87 | 50.96 | 9.48 | 27.59 | 46.70 | 32.87 | 27.59 |
| 8178700 | 116.24 | 157.23 | 69.32 | 109.52 | 135.50 | 61.32 | 101.02 | 130.30 | 109.52 | 101.02 |
| 8178700 | 3.20 | 11.94 | 0.00 | 0.09 | 2.38 | 0.00 | 0.00 | 1.45 | 0.09 | 0.00 |
| 8178700 | 0.15 | 15.24 | 0.00 | 0.50 | 4.10 | 0.00 | 0.11 | 2.89 | 0.50 | 0.11 |
| 8178700 | 30.52 | 136.14 | 51.74 | 88.44 | 113.85 | 46.52 | 82.07 | 109.71 | 51.74 | 46.52 |
| 8178700 | 3.47 | 24.38 | 0.00 | 3.25 | 10.41 | 0.00 | 1.89 | 8.31 | 3.25 | 1.89 |
| 8178700 | 0.71 | 20.07 | 0.00 | 1.70 | 7.24 | 0.00 | 0.81 | 5.54 | 1.70 | 0.81 |
| 8178700 | 0.00 | 3.05 | 0.00 | 0.00 | 0.00 | 0.00 | 0.00 | 0.00 | 0.00 | 0.00 |
| 8178700 | 0.23 | 39.62 | 1.41 | 10.23 | 22.15 | 0.83 | 8.19 | 19.86 | 1.41 | 0.83 |
| 8178700 | 0.00 | 1.27 | 0.00 | 0.00 | 0.00 | 0.00 | 0.00 | 0.00 | 0.00 | 0.00 |
| 8178700 | 0.03 | 9.40 | 0.00 | 0.00 | 1.03 | 0.00 | 0.00 | 0.62 | 0.00 | 0.00 |
| 8178700 | 0.02 | 5.84 | 0.00 | 0.00 | 0.16 | 0.00 | 0.00 | 0.02 | 0.00 | 0.00 |
| 8178700 | 0.00 | 15.24 | 0.00 | 0.38 | 3.80 | 0.00 | 0.11 | 2.89 | 0.38 | 0.11 |
| 8178700 | 0.01 | 5.59 | 0.00 | 0.00 | 0.13 | 0.00 | 0.00 | 0.01 | 0.00 | 0.00 |
| 8178700 | 0.01 | 6.35 | 0.00 | 0.00 | 0.26 | 0.00 | 0.00 | 0.06 | 0.00 | 0.00 |
| 8178700 | 0.76 | 33.02 | 0.44 | 6.71 | 16.68 | 0.15 | 5.06 | 14.60 | 0.44 | 0.15 |
| 9419740 | 0.43 | 8.13 | 0.00 | 0.00 | 0.00 | 0.00 | 0.00 | 0.00 | 0.00 | 0.00 |
| 9419740 | 0.68 | 5.08 | 0.00 | 0.00 | 0.00 | 0.00 | 0.00 | 0.00 | 0.00 | 0.00 |
| 9419740 | 1.21 | 4.06 | 0.00 | 0.00 | 0.00 | 0.00 | 0.00 | 0.00 | 0.00 | 0.00 |
| 9419740 | 0.00 | 1.02 | 0.00 | 0.00 | 0.00 | 0.00 | 0.00 | 0.00 | 0.00 | 0.00 |
| 9419740 | 0.27 | 7.11 | 0.00 | 0.00 | 0.00 | 0.00 | 0.00 | 0.00 | 0.00 | 0.00 |
| 9419740 | 0.15 | 7.11 | 0.00 | 0.00 | 0.00 | 0.00 | 0.00 | 0.00 | 0.00 | 0.00 |
| 9419740 | 0.00 | 1.02 | 0.00 | 0.00 | 0.00 | 0.00 | 0.00 | 0.00 | 0.00 | 0.00 |
| 9419740 | 4.20 | 11.18 | 0.00 | 0.00 | 0.25 | 0.00 | 0.00 | 0.12 | 0.00 | 0.00 |
| 9419740 | 10.62 | 23.37 | 0.00 | 0.19 | 4.34 | 0.00 | 0.08 | 3.62 | 0.19 | 0.08 |
| 9419740 | 0.00 | 1.02 | 0.00 | 0.00 | 0.00 | 0.00 | 0.00 | 0.00 | 0.00 | 0.00 |
| 9419740 | 0.85 | 10.16 | 0.00 | 0.00 | 0.11 | 0.00 | 0.00 | 0.04 | 0.00 | 0.00 |
| 9419740 | 0.05 | 7.11 | 0.00 | 0.00 | 0.00 | 0.00 | 0.00 | 0.00 | 0.00 | 0.00 |
| 9419740 | 0.00 | 2.03 | 0.00 | 0.00 | 0.00 | 0.00 | 0.00 | 0.00 | 0.00 | 0.00 |
| 9419740 | 22.34 | 22.35 | 0.00 | 0.09 | 3.76 | 0.00 | 0.03 | 3.19 | 3.76 | 3.19 |
| 9419740 | 0.17 | 5.08 | 0.00 | 0.00 | 0.00 | 0.00 | 0.00 | 0.00 | 0.00 | 0.00 |
| 9419740 | 0.02 | 4.06 | 0.00 | 0.00 | 0.00 | 0.00 | 0.00 | 0.00 | 0.00 | 0.00 |
| 9419740 | 0.00 | 4.06 | 0.00 | 0.00 | 0.00 | 0.00 | 0.00 | 0.00 | 0.00 | 0.00 |
| 9419740 | 0.15 | 2.03 | 0.00 | 0.00 | 0.00 | 0.00 | 0.00 | 0.00 | 0.00 | 0.00 |
| 9419740 | 1.65 | 17.27 | 0.00 | 0.00 | 1.64 | 0.00 | 0.00 | 1.37 | 0.00 | 0.00 |
| 9419740 | 5.73 | 12.19 | 0.00 | 0.00 | 0.42 | 0.00 | 0.00 | 0.24 | 0.00 | 0.00 |
| 9419740 | 0.22 | 23.37 | 0.00 | 0.13 | 4.09 | 0.00 | 0.08 | 3.62 | 0.00 | 0.00 |
| 9419740 | 14.01 | 34.54 | 0.00 | 1.95 | 10.29 | 0.00 | 1.69 | 9.50 | 1.95 | 1.69 |
| 9419740 | 0.00 | 1.02 | 0.00 | 0.00 | 0.00 | 0.00 | 0.00 | 0.00 | 0.00 | 0.00 |

**Supplementary Table 4.** Detailed information of randomly selected validation data for forest-dominated watersheds.

| Station | Q mm | P mm | Q CUSCN30 I | Q CUSCN30 II | Q CUSCN30 III | Q GCN I | Q GCN II | Q GCN III | Q CUSCN30 | Q GCN |
| --- | --- | --- | --- | --- | --- | --- | --- | --- | --- | --- |
| 2111180 | 0.08 | 5.08 | 0.00 | 0.00 | 0.00 | 0.00 | 0.00 | 0.00 | 0.00 | 0.00 |
| 2111180 | 0.07 | 1.02 | 0.00 | 0.00 | 0.00 | 0.00 | 0.00 | 0.00 | 0.00 | 0.00 |
| 2111180 | 9.55 | 34.80 | 0.00 | 0.00 | 3.83 | 0.00 | 2.22 | 10.84 | 0.00 | 2.22 |
| 2111180 | 0.67 | 14.99 | 0.00 | 0.00 | 0.00 | 0.00 | 0.00 | 1.06 | 0.00 | 0.00 |
| 2111180 | 17.38 | 80.26 | 0.02 | 9.23 | 29.56 | 5.32 | 23.90 | 46.69 | 9.23 | 23.90 |
| 2111180 | 0.00 | 3.81 | 0.00 | 0.00 | 0.00 | 0.00 | 0.00 | 0.00 | 0.00 | 0.00 |
| 2111180 | 0.00 | 3.81 | 0.00 | 0.00 | 0.00 | 0.00 | 0.00 | 0.00 | 0.00 | 0.00 |
| 2111180 | 2.49 | 4.57 | 0.00 | 0.00 | 0.00 | 0.00 | 0.00 | 0.00 | 0.00 | 0.00 |
| 2111180 | 44.91 | 85.34 | 0.18 | 11.37 | 33.49 | 6.76 | 27.15 | 51.16 | 11.37 | 27.15 |
| 2111180 | 4.18 | 50.29 | 0.00 | 1.26 | 10.99 | 0.21 | 7.69 | 21.86 | 1.26 | 7.69 |
| 2111180 | 0.03 | 9.91 | 0.00 | 0.00 | 0.00 | 0.00 | 0.00 | 0.09 | 0.00 | 0.00 |
| 2111180 | 1.29 | 27.94 | 0.00 | 0.00 | 1.79 | 0.00 | 0.75 | 6.70 | 0.00 | 0.75 |
| 2111180 | 0.24 | 7.11 | 0.00 | 0.00 | 0.00 | 0.00 | 0.00 | 0.00 | 0.00 | 0.00 |
| 2111180 | 4.29 | 80.01 | 0.02 | 9.17 | 29.42 | 5.25 | 23.74 | 46.47 | 0.02 | 5.25 |
| 2111180 | 0.10 | 12.70 | 0.00 | 0.00 | 0.00 | 0.00 | 0.00 | 0.51 | 0.00 | 0.00 |
| 2111180 | 0.13 | 22.35 | 0.00 | 0.00 | 0.57 | 0.00 | 0.11 | 3.84 | 0.00 | 0.11 |
| 2111180 | 4.19 | 23.88 | 0.00 | 0.00 | 0.86 | 0.00 | 0.23 | 4.56 | 0.00 | 0.23 |
| 2111180 | 0.06 | 8.89 | 0.00 | 0.00 | 0.00 | 0.00 | 0.00 | 0.02 | 0.00 | 0.00 |
| 2111180 | 3.29 | 81.79 | 0.04 | 9.75 | 30.57 | 5.74 | 24.86 | 48.02 | 0.04 | 5.74 |
| 2111180 | 0.05 | 2.03 | 0.00 | 0.00 | 0.00 | 0.00 | 0.00 | 0.00 | 0.00 | 0.00 |
| 2111180 | 0.59 | 19.30 | 0.00 | 0.00 | 0.20 | 0.00 | 0.00 | 2.53 | 0.00 | 0.00 |
| 2111180 | 1.83 | 31.50 | 0.00 | 0.00 | 2.84 | 0.00 | 1.43 | 8.77 | 0.00 | 1.43 |
| 2111180 | 0.14 | 29.46 | 0.00 | 0.00 | 2.12 | 0.00 | 1.02 | 7.56 | 0.00 | 1.02 |
| 2111180 | 0.00 | 0.51 | 0.00 | 0.00 | 0.00 | 0.00 | 0.00 | 0.00 | 0.00 | 0.00 |
| 2111180 | 0.57 | 28.70 | 0.00 | 0.00 | 1.93 | 0.00 | 0.88 | 7.13 | 0.00 | 0.88 |
| 2111180 | 0.66 | 14.22 | 0.00 | 0.00 | 0.00 | 0.00 | 0.00 | 0.86 | 0.00 | 0.00 |
| 2111180 | 0.15 | 0.51 | 0.00 | 0.00 | 0.00 | 0.00 | 0.00 | 0.00 | 0.00 | 0.00 |
| 2111180 | 2.77 | 40.89 | 0.00 | 0.18 | 6.27 | 0.00 | 4.06 | 14.94 | 0.18 | 4.06 |
| 2111180 | 0.46 | 22.86 | 0.00 | 0.00 | 0.68 | 0.00 | 0.14 | 4.07 | 0.00 | 0.14 |
| 2111180 | 0.00 | 1.02 | 0.00 | 0.00 | 0.00 | 0.00 | 0.00 | 0.00 | 0.00 | 0.00 |
| 2111180 | 0.40 | 21.08 | 0.00 | 0.00 | 0.39 | 0.00 | 0.04 | 3.27 | 0.00 | 0.04 |
| 2111180 | 1.71 | 35.56 | 0.00 | 0.00 | 4.17 | 0.00 | 2.43 | 11.33 | 0.00 | 2.43 |
| 2111180 | 0.27 | 21.34 | 0.00 | 0.00 | 0.44 | 0.00 | 0.05 | 3.38 | 0.00 | 0.05 |
| 2111180 | 26.87 | 103.89 | 1.69 | 19.40 | 47.24 | 13.13 | 39.90 | 67.86 | 19.40 | 39.90 |
| 2111180 | 0.01 | 0.51 | 0.00 | 0.00 | 0.00 | 0.00 | 0.00 | 0.00 | 0.00 | 0.00 |
| 2111180 | 0.25 | 18.03 | 0.00 | 0.00 | 0.08 | 0.00 | 0.00 | 2.05 | 0.00 | 0.00 |
| 2111180 | 0.04 | 4.06 | 0.00 | 0.00 | 0.00 | 0.00 | 0.00 | 0.00 | 0.00 | 0.00 |
| 2111180 | 0.07 | 7.11 | 0.00 | 0.00 | 0.00 | 0.00 | 0.00 | 0.00 | 0.00 | 0.00 |
| 2111180 | 0.32 | 26.67 | 0.00 | 0.00 | 1.40 | 0.00 | 0.56 | 6.00 | 0.00 | 0.56 |
| 2111180 | 0.04 | 6.35 | 0.00 | 0.00 | 0.00 | 0.00 | 0.00 | 0.00 | 0.00 | 0.00 |
| 2111180 | 0.10 | 15.24 | 0.00 | 0.00 | 0.00 | 0.00 | 0.00 | 1.13 | 0.00 | 0.00 |
| 2111180 | 0.00 | 2.54 | 0.00 | 0.00 | 0.00 | 0.00 | 0.00 | 0.00 | 0.00 | 0.00 |
| 2111180 | 0.93 | 30.99 | 0.00 | 0.00 | 2.67 | 0.00 | 1.32 | 8.46 | 0.00 | 1.32 |
| 2111180 | 0.61 | 31.75 | 0.00 | 0.00 | 2.89 | 0.00 | 1.48 | 8.92 | 0.00 | 1.48 |
| 2111180 | 0.64 | 23.88 | 0.00 | 0.00 | 0.87 | 0.00 | 0.23 | 4.56 | 0.00 | 0.23 |
| 2111180 | 0.07 | 3.56 | 0.00 | 0.00 | 0.00 | 0.00 | 0.00 | 0.00 | 0.00 | 0.00 |
| 2111180 | 0.41 | 18.03 | 0.00 | 0.00 | 0.08 | 0.00 | 0.00 | 2.05 | 0.00 | 0.00 |
| 2111180 | 4.47 | 34.54 | 0.00 | 0.00 | 3.79 | 0.00 | 2.15 | 10.67 | 0.00 | 2.15 |
| 2111180 | 0.03 | 1.52 | 0.00 | 0.00 | 0.00 | 0.00 | 0.00 | 0.00 | 0.00 | 0.00 |
| 2111180 | 0.00 | 10.92 | 0.00 | 0.00 | 0.00 | 0.00 | 0.00 | 0.21 | 0.00 | 0.00 |
| 2111180 | 0.41 | 10.41 | 0.00 | 0.00 | 0.00 | 0.00 | 0.00 | 0.14 | 0.00 | 0.00 |
| 2111180 | 0.36 | 17.02 | 0.00 | 0.00 | 0.04 | 0.00 | 0.00 | 1.69 | 0.00 | 0.00 |
| 2111180 | 0.06 | 0.25 | 0.00 | 0.00 | 0.00 | 0.00 | 0.00 | 0.00 | 0.00 | 0.00 |
| 2111180 | 0.00 | 0.51 | 0.00 | 0.00 | 0.00 | 0.00 | 0.00 | 0.00 | 0.00 | 0.00 |
| 2111180 | 0.05 | 0.76 | 0.00 | 0.00 | 0.00 | 0.00 | 0.00 | 0.00 | 0.00 | 0.00 |
| 2111180 | 0.00 | 1.27 | 0.00 | 0.00 | 0.00 | 0.00 | 0.00 | 0.00 | 0.00 | 0.00 |
| 2111180 | 0.00 | 2.54 | 0.00 | 0.00 | 0.00 | 0.00 | 0.00 | 0.00 | 0.00 | 0.00 |
| 2111180 | 2.81 | 35.31 | 0.00 | 0.00 | 4.16 | 0.00 | 2.36 | 11.16 | 0.00 | 2.36 |
| 2111180 | 0.00 | 0.25 | 0.00 | 0.00 | 0.00 | 0.00 | 0.00 | 0.00 | 0.00 | 0.00 |
| 2111180 | 3.05 | 51.56 | 0.00 | 1.45 | 11.61 | 0.29 | 8.25 | 22.83 | 0.00 | 0.29 |
| 2111180 | 0.09 | 6.10 | 0.00 | 0.00 | 0.00 | 0.00 | 0.00 | 0.00 | 0.00 | 0.00 |
| 2111180 | 0.15 | 12.45 | 0.00 | 0.00 | 0.00 | 0.00 | 0.00 | 0.46 | 0.00 | 0.00 |
| 2111180 | 0.39 | 2.54 | 0.00 | 0.00 | 0.00 | 0.00 | 0.00 | 0.00 | 0.00 | 0.00 |
| 2111180 | 0.00 | 0.76 | 0.00 | 0.00 | 0.00 | 0.00 | 0.00 | 0.00 | 0.00 | 0.00 |
| 2111180 | 1.50 | 38.35 | 0.00 | 0.06 | 5.26 | 0.00 | 3.24 | 13.19 | 0.06 | 3.24 |
| 2111180 | 0.00 | 4.83 | 0.00 | 0.00 | 0.00 | 0.00 | 0.00 | 0.00 | 0.00 | 0.00 |
| 2111180 | 0.03 | 9.14 | 0.00 | 0.00 | 0.00 | 0.00 | 0.00 | 0.04 | 0.00 | 0.00 |
| 2111180 | 0.31 | 10.67 | 0.00 | 0.00 | 0.00 | 0.00 | 0.00 | 0.18 | 0.00 | 0.00 |
| 2111180 | 6.80 | 43.69 | 0.00 | 0.43 | 7.69 | 0.00 | 5.04 | 16.93 | 0.43 | 5.04 |
| 2111180 | 0.00 | 0.25 | 0.00 | 0.00 | 0.00 | 0.00 | 0.00 | 0.00 | 0.00 | 0.00 |
| 2111180 | 0.08 | 6.10 | 0.00 | 0.00 | 0.00 | 0.00 | 0.00 | 0.00 | 0.00 | 0.00 |
| 2111180 | 0.00 | 6.35 | 0.00 | 0.00 | 0.00 | 0.00 | 0.00 | 0.00 | 0.00 | 0.00 |
| 2111180 | 2.44 | 19.30 | 0.00 | 0.00 | 0.20 | 0.00 | 0.00 | 2.53 | 0.00 | 0.00 |
| 2111180 | 0.07 | 14.48 | 0.00 | 0.00 | 0.00 | 0.00 | 0.00 | 0.92 | 0.00 | 0.00 |
| 2111180 | 1.95 | 31.75 | 0.00 | 0.00 | 2.82 | 0.00 | 1.48 | 8.92 | 0.00 | 1.48 |
| 2111180 | 0.50 | 23.11 | 0.00 | 0.00 | 0.68 | 0.00 | 0.16 | 4.19 | 0.00 | 0.16 |
| 2111180 | 0.00 | 0.51 | 0.00 | 0.00 | 0.00 | 0.00 | 0.00 | 0.00 | 0.00 | 0.00 |
| 2111180 | 0.62 | 23.11 | 0.00 | 0.00 | 0.73 | 0.00 | 0.16 | 4.19 | 0.00 | 0.16 |
| 2111180 | 0.26 | 11.68 | 0.00 | 0.00 | 0.00 | 0.00 | 0.00 | 0.32 | 0.00 | 0.00 |
| 2111180 | 5.67 | 74.17 | 0.00 | 7.06 | 25.29 | 3.79 | 20.16 | 41.40 | 0.00 | 3.79 |
| 2111180 | 19.01 | 67.82 | 0.00 | 5.22 | 21.31 | 2.45 | 16.47 | 36.00 | 5.22 | 16.47 |
| 2111180 | 0.58 | 22.35 | 0.00 | 0.00 | 0.59 | 0.00 | 0.11 | 3.84 | 0.00 | 0.11 |
| 2111180 | 0.00 | 9.14 | 0.00 | 0.00 | 0.00 | 0.00 | 0.00 | 0.04 | 0.00 | 0.00 |
| 2111180 | 23.56 | 215.39 | 36.70 | 91.93 | 144.74 | 75.91 | 132.09 | 174.12 | 36.70 | 75.91 |
| 2111180 | 0.25 | 30.23 | 0.00 | 0.00 | 2.42 | 0.00 | 1.16 | 8.01 | 0.00 | 1.16 |
| 2111180 | 1.33 | 22.86 | 0.00 | 0.00 | 0.67 | 0.00 | 0.14 | 4.07 | 0.00 | 0.14 |
| 2111180 | 0.35 | 21.59 | 0.00 | 0.00 | 0.45 | 0.00 | 0.06 | 3.49 | 0.00 | 0.06 |
| 2111180 | 0.00 | 0.51 | 0.00 | 0.00 | 0.00 | 0.00 | 0.00 | 0.00 | 0.00 | 0.00 |
| 2111180 | 0.56 | 26.42 | 0.00 | 0.00 | 1.37 | 0.00 | 0.52 | 5.87 | 0.00 | 0.52 |
| 2111180 | 12.21 | 75.95 | 0.00 | 7.88 | 26.84 | 4.22 | 21.23 | 42.93 | 7.88 | 21.23 |
| 2111180 | 0.03 | 2.03 | 0.00 | 0.00 | 0.00 | 0.00 | 0.00 | 0.00 | 0.00 | 0.00 |
| 2111180 | 0.03 | 25.40 | 0.00 | 0.00 | 1.18 | 0.00 | 0.39 | 5.33 | 0.00 | 0.39 |
| 2111180 | 0.41 | 21.34 | 0.00 | 0.00 | 0.42 | 0.00 | 0.05 | 3.38 | 0.00 | 0.05 |
| 2111180 | 0.06 | 5.59 | 0.00 | 0.00 | 0.00 | 0.00 | 0.00 | 0.00 | 0.00 | 0.00 |
| 3456500 | 41.89 | 81.53 | 0.00 | 5.05 | 23.00 | 3.71 | 20.81 | 43.67 | 5.05 | 20.81 |
| 3456500 | 13.93 | 45.21 | 0.00 | 0.00 | 4.89 | 0.00 | 4.01 | 15.48 | 0.00 | 4.01 |
| 3456500 | 17.26 | 49.28 | 0.00 | 0.06 | 6.32 | 0.00 | 5.37 | 18.30 | 0.06 | 5.37 |
| 3456500 | 0.20 | 4.57 | 0.00 | 0.00 | 0.00 | 0.00 | 0.00 | 0.00 | 0.00 | 0.00 |
| 3456500 | 0.00 | 2.29 | 0.00 | 0.00 | 0.00 | 0.00 | 0.00 | 0.00 | 0.00 | 0.00 |
| 3456500 | 0.00 | 2.03 | 0.00 | 0.00 | 0.00 | 0.00 | 0.00 | 0.00 | 0.00 | 0.00 |
| 3456500 | 0.39 | 25.91 | 0.00 | 0.00 | 0.29 | 0.00 | 0.13 | 4.29 | 0.00 | 0.13 |
| 3456500 | 0.25 | 19.81 | 0.00 | 0.00 | 0.00 | 0.00 | 0.00 | 1.89 | 0.00 | 0.00 |
| 3456500 | 44.65 | 148.08 | 3.65 | 31.97 | 70.82 | 28.21 | 67.15 | 103.56 | 3.65 | 28.21 |
| 3456500 | 24.70 | 47.75 | 0.00 | 0.09 | 6.41 | 0.00 | 4.84 | 17.23 | 0.09 | 4.84 |
| 3456500 | 0.41 | 17.27 | 0.00 | 0.00 | 0.00 | 0.00 | 0.00 | 1.13 | 0.00 | 0.00 |
| 3456500 | 0.33 | 7.11 | 0.00 | 0.00 | 0.00 | 0.00 | 0.00 | 0.00 | 0.00 | 0.00 |
| 3456500 | 0.00 | 4.06 | 0.00 | 0.00 | 0.00 | 0.00 | 0.00 | 0.00 | 0.00 | 0.00 |
| 3456500 | 1.87 | 21.34 | 0.00 | 0.00 | 0.01 | 0.00 | 0.00 | 2.42 | 0.00 | 0.00 |
| 3456500 | 0.06 | 2.29 | 0.00 | 0.00 | 0.00 | 0.00 | 0.00 | 0.00 | 0.00 | 0.00 |
| 3456500 | 0.00 | 0.25 | 0.00 | 0.00 | 0.00 | 0.00 | 0.00 | 0.00 | 0.00 | 0.00 |
| 3456500 | 0.66 | 9.14 | 0.00 | 0.00 | 0.00 | 0.00 | 0.00 | 0.00 | 0.00 | 0.00 |
| 3456500 | 0.95 | 40.64 | 0.00 | 0.00 | 3.32 | 0.00 | 2.67 | 12.47 | 0.00 | 0.00 |
| 3456500 | 0.00 | 0.51 | 0.00 | 0.00 | 0.00 | 0.00 | 0.00 | 0.00 | 0.00 | 0.00 |
| 3456500 | 0.53 | 34.04 | 0.00 | 0.00 | 1.65 | 0.00 | 1.17 | 8.47 | 0.00 | 0.00 |
| 3456500 | 2.11 | 22.86 | 0.00 | 0.00 | 0.05 | 0.00 | 0.01 | 2.99 | 0.00 | 0.01 |
| 3456500 | 0.02 | 4.06 | 0.00 | 0.00 | 0.00 | 0.00 | 0.00 | 0.00 | 0.00 | 0.00 |
| 3456500 | 0.00 | 0.51 | 0.00 | 0.00 | 0.00 | 0.00 | 0.00 | 0.00 | 0.00 | 0.00 |
| 3456500 | 2.30 | 6.35 | 0.00 | 0.00 | 0.00 | 0.00 | 0.00 | 0.00 | 0.00 | 0.00 |
| 3456500 | 0.20 | 8.64 | 0.00 | 0.00 | 0.00 | 0.00 | 0.00 | 0.00 | 0.00 | 0.00 |
| 3456500 | 7.86 | 19.05 | 0.00 | 0.00 | 0.00 | 0.00 | 0.00 | 1.64 | 0.00 | 0.00 |
| 3456500 | 0.00 | 0.51 | 0.00 | 0.00 | 0.00 | 0.00 | 0.00 | 0.00 | 0.00 | 0.00 |
| 3456500 | 0.67 | 24.38 | 0.00 | 0.00 | 0.15 | 0.00 | 0.05 | 3.62 | 0.00 | 0.05 |
| 3456500 | 0.00 | 2.79 | 0.00 | 0.00 | 0.00 | 0.00 | 0.00 | 0.00 | 0.00 | 0.00 |
| 3456500 | 0.00 | 9.40 | 0.00 | 0.00 | 0.00 | 0.00 | 0.00 | 0.00 | 0.00 | 0.00 |
| 3456500 | 0.00 | 0.51 | 0.00 | 0.00 | 0.00 | 0.00 | 0.00 | 0.00 | 0.00 | 0.00 |
| 3456500 | 0.00 | 9.65 | 0.00 | 0.00 | 0.00 | 0.00 | 0.00 | 0.00 | 0.00 | 0.00 |
| 3456500 | 0.07 | 0.76 | 0.00 | 0.00 | 0.00 | 0.00 | 0.00 | 0.00 | 0.00 | 0.00 |
| 3456500 | 0.00 | 3.81 | 0.00 | 0.00 | 0.00 | 0.00 | 0.00 | 0.00 | 0.00 | 0.00 |
| 3456500 | 0.03 | 19.56 | 0.00 | 0.00 | 0.00 | 0.00 | 0.00 | 1.80 | 0.00 | 0.00 |
| 3456500 | 0.02 | 7.87 | 0.00 | 0.00 | 0.00 | 0.00 | 0.00 | 0.00 | 0.00 | 0.00 |
| 3456500 | 0.13 | 4.32 | 0.00 | 0.00 | 0.00 | 0.00 | 0.00 | 0.00 | 0.00 | 0.00 |
| 3456500 | 2.44 | 14.99 | 0.00 | 0.00 | 0.00 | 0.00 | 0.00 | 0.59 | 0.00 | 0.00 |
| 3456500 | 0.95 | 8.89 | 0.00 | 0.00 | 0.00 | 0.00 | 0.00 | 0.00 | 0.00 | 0.00 |
| 3456500 | 0.00 | 1.78 | 0.00 | 0.00 | 0.00 | 0.00 | 0.00 | 0.00 | 0.00 | 0.00 |
| 3456500 | 1.08 | 4.57 | 0.00 | 0.00 | 0.00 | 0.00 | 0.00 | 0.00 | 0.00 | 0.00 |
| 3456500 | 1.72 | 8.13 | 0.00 | 0.00 | 0.00 | 0.00 | 0.00 | 0.00 | 0.00 | 0.00 |
| 3456500 | 0.97 | 16.26 | 0.00 | 0.00 | 0.00 | 0.00 | 0.00 | 0.87 | 0.00 | 0.00 |
| 3456500 | 0.12 | 17.27 | 0.00 | 0.00 | 0.00 | 0.00 | 0.00 | 1.13 | 0.00 | 0.00 |
| 3456500 | 3.60 | 33.27 | 0.00 | 0.00 | 1.73 | 0.00 | 1.03 | 8.04 | 0.00 | 1.03 |
| 3456500 | 2.16 | 39.88 | 0.00 | 0.00 | 3.06 | 0.00 | 2.47 | 11.98 | 0.00 | 0.00 |
| 3456500 | 6.31 | 30.73 | 0.00 | 0.00 | 0.92 | 0.00 | 0.63 | 6.66 | 0.00 | 0.63 |
| 3456500 | 0.01 | 0.51 | 0.00 | 0.00 | 0.00 | 0.00 | 0.00 | 0.00 | 0.00 | 0.00 |
| 3456500 | 9.12 | 18.29 | 0.00 | 0.00 | 0.00 | 0.00 | 0.00 | 1.41 | 0.00 | 0.00 |
| 3456500 | 0.79 | 13.97 | 0.00 | 0.00 | 0.00 | 0.00 | 0.00 | 0.40 | 0.00 | 0.00 |
| 3456500 | 5.16 | 48.77 | 0.00 | 0.05 | 6.13 | 0.00 | 5.19 | 17.94 | 0.00 | 0.00 |
| 3456500 | 0.16 | 5.59 | 0.00 | 0.00 | 0.00 | 0.00 | 0.00 | 0.00 | 0.00 | 0.00 |
| 3456500 | 1.48 | 3.05 | 0.00 | 0.00 | 0.00 | 0.00 | 0.00 | 0.00 | 0.00 | 0.00 |
| 3456500 | 0.00 | 4.57 | 0.00 | 0.00 | 0.00 | 0.00 | 0.00 | 0.00 | 0.00 | 0.00 |
| 3456500 | 1.79 | 22.35 | 0.00 | 0.00 | 0.03 | 0.00 | 0.00 | 2.80 | 0.00 | 0.00 |
| 3456500 | 1.81 | 17.78 | 0.00 | 0.00 | 0.00 | 0.00 | 0.00 | 1.27 | 0.00 | 0.00 |
| 3456500 | 3.53 | 37.85 | 0.00 | 0.00 | 2.49 | 0.00 | 1.97 | 10.72 | 0.00 | 1.97 |
| 3456500 | 6.44 | 62.74 | 0.00 | 1.40 | 12.83 | 0.69 | 10.94 | 28.35 | 0.00 | 0.69 |
| 3456500 | 0.01 | 0.76 | 0.00 | 0.00 | 0.00 | 0.00 | 0.00 | 0.00 | 0.00 | 0.00 |
| 3456500 | 0.16 | 11.94 | 0.00 | 0.00 | 0.00 | 0.00 | 0.00 | 0.13 | 0.00 | 0.00 |
| 3456500 | 0.00 | 1.02 | 0.00 | 0.00 | 0.00 | 0.00 | 0.00 | 0.00 | 0.00 | 0.00 |
| 3456500 | 11.26 | 50.80 | 0.00 | 0.12 | 6.87 | 0.01 | 5.93 | 19.38 | 0.12 | 5.93 |
| 3456500 | 0.33 | 12.45 | 0.00 | 0.00 | 0.00 | 0.00 | 0.00 | 0.18 | 0.00 | 0.00 |
| 3456500 | 0.11 | 0.25 | 0.00 | 0.00 | 0.00 | 0.00 | 0.00 | 0.00 | 0.00 | 0.00 |
| 3456500 | 0.06 | 1.52 | 0.00 | 0.00 | 0.00 | 0.00 | 0.00 | 0.00 | 0.00 | 0.00 |
| 3456500 | 0.10 | 7.37 | 0.00 | 0.00 | 0.00 | 0.00 | 0.00 | 0.00 | 0.00 | 0.00 |
| 3456500 | 0.69 | 10.67 | 0.00 | 0.00 | 0.00 | 0.00 | 0.00 | 0.03 | 0.00 | 0.00 |
| 3456500 | 0.56 | 32.00 | 0.00 | 0.00 | 1.17 | 0.00 | 0.82 | 7.34 | 0.00 | 0.00 |
| 3456500 | 0.38 | 15.24 | 0.00 | 0.00 | 0.00 | 0.00 | 0.00 | 0.64 | 0.00 | 0.00 |
| 3456500 | 0.47 | 26.92 | 0.00 | 0.00 | 0.41 | 0.00 | 0.20 | 4.76 | 0.00 | 0.00 |
| 3456500 | 0.16 | 4.32 | 0.00 | 0.00 | 0.00 | 0.00 | 0.00 | 0.00 | 0.00 | 0.00 |
| 3456500 | 0.00 | 10.41 | 0.00 | 0.00 | 0.00 | 0.00 | 0.00 | 0.02 | 0.00 | 0.00 |
| 3456500 | 0.00 | 1.78 | 0.00 | 0.00 | 0.00 | 0.00 | 0.00 | 0.00 | 0.00 | 0.00 |
| 3456500 | 0.12 | 11.18 | 0.00 | 0.00 | 0.00 | 0.00 | 0.00 | 0.06 | 0.00 | 0.00 |
| 3456500 | 0.07 | 2.79 | 0.00 | 0.00 | 0.00 | 0.00 | 0.00 | 0.00 | 0.00 | 0.00 |
| 3456500 | 2.39 | 13.46 | 0.00 | 0.00 | 0.00 | 0.00 | 0.00 | 0.32 | 0.00 | 0.00 |
| 3456500 | 0.00 | 8.89 | 0.00 | 0.00 | 0.00 | 0.00 | 0.00 | 0.00 | 0.00 | 0.00 |
| 3456500 | 3.70 | 48.51 | 0.00 | 0.04 | 5.98 | 0.00 | 5.10 | 17.76 | 0.00 | 0.00 |
| 3456500 | 1.04 | 5.33 | 0.00 | 0.00 | 0.00 | 0.00 | 0.00 | 0.00 | 0.00 | 0.00 |
| 3456500 | 0.92 | 42.16 | 0.00 | 0.00 | 4.32 | 0.00 | 3.09 | 13.45 | 0.00 | 0.00 |
| 3456500 | 4.78 | 38.10 | 0.00 | 0.00 | 2.60 | 0.00 | 2.03 | 10.88 | 0.00 | 2.03 |
| 3456500 | 0.00 | 2.79 | 0.00 | 0.00 | 0.00 | 0.00 | 0.00 | 0.00 | 0.00 | 0.00 |
| 3456500 | 1.82 | 26.16 | 0.00 | 0.00 | 0.32 | 0.00 | 0.15 | 4.40 | 0.00 | 0.15 |
| 3456500 | 0.00 | 3.30 | 0.00 | 0.00 | 0.00 | 0.00 | 0.00 | 0.00 | 0.00 | 0.00 |
| 3456500 | 0.25 | 4.57 | 0.00 | 0.00 | 0.00 | 0.00 | 0.00 | 0.00 | 0.00 | 0.00 |
| 3456500 | 0.05 | 1.27 | 0.00 | 0.00 | 0.00 | 0.00 | 0.00 | 0.00 | 0.00 | 0.00 |
| 3456500 | 0.43 | 13.97 | 0.00 | 0.00 | 0.00 | 0.00 | 0.00 | 0.40 | 0.00 | 0.00 |
| 3456500 | 0.03 | 3.81 | 0.00 | 0.00 | 0.00 | 0.00 | 0.00 | 0.00 | 0.00 | 0.00 |
| 3456500 | 0.00 | 1.27 | 0.00 | 0.00 | 0.00 | 0.00 | 0.00 | 0.00 | 0.00 | 0.00 |
| 3456500 | 0.00 | 0.76 | 0.00 | 0.00 | 0.00 | 0.00 | 0.00 | 0.00 | 0.00 | 0.00 |
| 3456500 | 8.34 | 41.66 | 0.00 | 0.00 | 3.72 | 0.00 | 2.95 | 13.12 | 0.00 | 2.95 |
| 3456500 | 3.41 | 27.69 | 0.00 | 0.00 | 0.48 | 0.00 | 0.27 | 5.12 | 0.00 | 0.27 |
| 3456500 | 0.36 | 6.35 | 0.00 | 0.00 | 0.00 | 0.00 | 0.00 | 0.00 | 0.00 | 0.00 |
| 3456500 | 1.44 | 33.02 | 0.00 | 0.00 | 1.37 | 0.00 | 0.99 | 7.90 | 0.00 | 0.99 |
| 3479000 | 0.22 | 11.68 | 0.00 | 0.00 | 0.00 | 0.00 | 0.00 | 0.39 | 0.00 | 0.00 |
| 3479000 | 0.84 | 30.99 | 0.00 | 0.00 | 0.98 | 0.00 | 1.50 | 8.85 | 0.00 | 1.50 |
| 3479000 | 1.30 | 92.46 | 0.00 | 7.64 | 29.04 | 9.75 | 32.93 | 58.46 | 0.00 | 9.75 |
| 3479000 | 0.00 | 1.78 | 0.00 | 0.00 | 0.00 | 0.00 | 0.00 | 0.00 | 0.00 | 0.00 |
| 3479000 | 0.68 | 57.15 | 0.00 | 0.48 | 9.31 | 1.01 | 11.43 | 27.94 | 0.00 | 1.01 |
| 3479000 | 0.26 | 275.84 | 41.58 | 114.04 | 180.61 | 123.09 | 189.46 | 234.74 | 41.58 | 123.09 |
| 3479000 | 0.00 | 6.60 | 0.00 | 0.00 | 0.00 | 0.00 | 0.00 | 0.00 | 0.00 | 0.00 |
| 3479000 | 0.64 | 27.18 | 0.00 | 0.00 | 0.41 | 0.00 | 0.75 | 6.61 | 0.00 | 0.75 |
| 3479000 | 0.34 | 13.21 | 0.00 | 0.00 | 0.00 | 0.00 | 0.00 | 0.71 | 0.00 | 0.00 |
| 3479000 | 0.00 | 2.29 | 0.00 | 0.00 | 0.00 | 0.00 | 0.00 | 0.00 | 0.00 | 0.00 |
| 3479000 | 83.87 | 150.88 | 4.02 | 33.28 | 72.90 | 37.11 | 78.04 | 113.06 | 33.28 | 78.04 |
| 3479000 | 0.00 | 8.89 | 0.00 | 0.00 | 0.00 | 0.00 | 0.00 | 0.04 | 0.00 | 0.00 |
| 3479000 | 1.97 | 23.37 | 0.00 | 0.00 | 0.08 | 0.00 | 0.25 | 4.58 | 0.00 | 0.25 |
| 3479000 | 1.51 | 20.57 | 0.00 | 0.00 | 0.00 | 0.00 | 0.05 | 3.27 | 0.00 | 0.05 |
| 3479000 | 0.00 | 4.06 | 0.00 | 0.00 | 0.00 | 0.00 | 0.00 | 0.00 | 0.00 | 0.00 |
| 3479000 | 0.00 | 3.30 | 0.00 | 0.00 | 0.00 | 0.00 | 0.00 | 0.00 | 0.00 | 0.00 |
| 3479000 | 0.38 | 45.97 | 0.00 | 0.00 | 4.84 | 0.07 | 6.32 | 19.19 | 0.00 | 0.07 |
| 3479000 | 0.28 | 16.26 | 0.00 | 0.00 | 0.00 | 0.00 | 0.00 | 1.58 | 0.00 | 0.00 |
| 3479000 | 0.42 | 20.57 | 0.00 | 0.00 | 0.00 | 0.00 | 0.05 | 3.27 | 0.00 | 0.05 |
| 3479000 | 0.01 | 15.49 | 0.00 | 0.00 | 0.00 | 0.00 | 0.00 | 1.34 | 0.00 | 0.00 |
| 3479000 | 0.62 | 10.41 | 0.00 | 0.00 | 0.00 | 0.00 | 0.00 | 0.19 | 0.00 | 0.00 |
| 3479000 | 0.15 | 42.67 | 0.00 | 0.00 | 3.73 | 0.00 | 5.03 | 16.75 | 0.00 | 0.00 |
| 3479000 | 0.13 | 23.62 | 0.00 | 0.00 | 0.07 | 0.00 | 0.27 | 4.71 | 0.00 | 0.27 |
| 3479000 | 1.11 | 21.84 | 0.00 | 0.00 | 0.02 | 0.00 | 0.12 | 3.85 | 0.00 | 0.12 |
| 3479000 | 0.33 | 7.11 | 0.00 | 0.00 | 0.00 | 0.00 | 0.00 | 0.00 | 0.00 | 0.00 |
| 3479000 | 27.05 | 116.08 | 0.32 | 16.73 | 46.14 | 19.27 | 50.18 | 80.17 | 16.73 | 50.18 |
| 3479000 | 0.30 | 2.03 | 0.00 | 0.00 | 0.00 | 0.00 | 0.00 | 0.00 | 0.00 | 0.00 |
| 3479000 | 0.00 | 5.33 | 0.00 | 0.00 | 0.00 | 0.00 | 0.00 | 0.00 | 0.00 | 0.00 |
| 3479000 | 2.73 | 20.83 | 0.00 | 0.00 | 0.00 | 0.00 | 0.06 | 3.38 | 0.00 | 0.06 |
| 3479000 | 85.27 | 210.82 | 18.67 | 69.63 | 123.73 | 75.14 | 130.00 | 171.00 | 18.67 | 75.14 |
| 3479000 | 0.00 | 10.41 | 0.00 | 0.00 | 0.00 | 0.00 | 0.00 | 0.19 | 0.00 | 0.00 |
| 3479000 | 0.00 | 16.00 | 0.00 | 0.00 | 0.00 | 0.00 | 0.00 | 1.50 | 0.00 | 0.00 |
| 3479000 | 0.73 | 1.78 | 0.00 | 0.00 | 0.00 | 0.00 | 0.00 | 0.00 | 0.00 | 0.00 |
| 3479000 | 0.00 | 1.02 | 0.00 | 0.00 | 0.00 | 0.00 | 0.00 | 0.00 | 0.00 | 0.00 |
| 3479000 | 30.43 | 70.61 | 0.00 | 2.45 | 16.35 | 3.42 | 18.83 | 39.18 | 2.45 | 18.83 |
| 3479000 | 2.13 | 3.56 | 0.00 | 0.00 | 0.00 | 0.00 | 0.00 | 0.00 | 0.00 | 0.00 |
| 3479000 | 1.50 | 43.94 | 0.00 | 0.00 | 4.31 | 0.02 | 5.51 | 17.68 | 0.00 | 5.51 |
| 3479000 | 9.24 | 20.83 | 0.00 | 0.00 | 0.00 | 0.00 | 0.06 | 3.38 | 0.00 | 0.06 |
| 3479000 | 0.00 | 0.25 | 0.00 | 0.00 | 0.00 | 0.00 | 0.00 | 0.00 | 0.00 | 0.00 |
| 3479000 | 6.59 | 47.50 | 0.00 | 0.02 | 5.61 | 0.14 | 6.95 | 20.34 | 0.02 | 6.95 |
| 3479000 | 0.66 | 13.72 | 0.00 | 0.00 | 0.00 | 0.00 | 0.00 | 0.83 | 0.00 | 0.00 |
| 3479000 | 1.19 | 31.50 | 0.00 | 0.00 | 0.98 | 0.00 | 1.61 | 9.16 | 0.00 | 1.61 |
| 3479000 | 37.21 | 51.82 | 0.00 | 0.17 | 7.34 | 0.43 | 8.86 | 23.68 | 0.17 | 8.86 |
| 3479000 | 0.05 | 7.87 | 0.00 | 0.00 | 0.00 | 0.00 | 0.00 | 0.00 | 0.00 | 0.00 |
| 3479000 | 0.20 | 39.12 | 0.00 | 0.00 | 3.18 | 0.00 | 3.78 | 14.21 | 0.00 | 3.78 |
| 3479000 | 0.00 | 24.13 | 0.00 | 0.00 | 0.19 | 0.00 | 0.33 | 4.97 | 0.00 | 0.33 |
| 3479000 | 17.71 | 92.20 | 0.00 | 7.87 | 29.37 | 9.66 | 32.76 | 58.23 | 7.87 | 32.76 |
| 3479000 | 0.00 | 43.43 | 0.00 | 0.00 | 4.20 | 0.01 | 5.32 | 17.31 | 0.00 | 0.01 |
| 3479000 | 0.00 | 11.18 | 0.00 | 0.00 | 0.00 | 0.00 | 0.00 | 0.30 | 0.00 | 0.00 |
| 3479000 | 0.21 | 13.46 | 0.00 | 0.00 | 0.00 | 0.00 | 0.00 | 0.77 | 0.00 | 0.00 |
| 3479000 | 0.00 | 1.02 | 0.00 | 0.00 | 0.00 | 0.00 | 0.00 | 0.00 | 0.00 | 0.00 |
| 3479000 | 0.00 | 4.32 | 0.00 | 0.00 | 0.00 | 0.00 | 0.00 | 0.00 | 0.00 | 0.00 |
| 3479000 | 0.40 | 15.49 | 0.00 | 0.00 | 0.00 | 0.00 | 0.00 | 1.34 | 0.00 | 0.00 |
| 3479000 | 0.00 | 3.81 | 0.00 | 0.00 | 0.00 | 0.00 | 0.00 | 0.00 | 0.00 | 0.00 |
| 3479000 | 0.09 | 2.54 | 0.00 | 0.00 | 0.00 | 0.00 | 0.00 | 0.00 | 0.00 | 0.00 |
| 3479000 | 0.00 | 34.04 | 0.00 | 0.00 | 1.77 | 0.00 | 2.25 | 10.78 | 0.00 | 0.00 |
| 3479000 | 3.70 | 23.37 | 0.00 | 0.00 | 0.08 | 0.00 | 0.25 | 4.58 | 0.00 | 0.25 |
| 3479000 | 0.28 | 18.03 | 0.00 | 0.00 | 0.00 | 0.00 | 0.00 | 2.22 | 0.00 | 0.00 |
| 3479000 | 0.02 | 1.02 | 0.00 | 0.00 | 0.00 | 0.00 | 0.00 | 0.00 | 0.00 | 0.00 |
| 3479000 | 0.00 | 32.77 | 0.00 | 0.00 | 1.20 | 0.00 | 1.92 | 9.96 | 0.00 | 1.92 |
| 3479000 | 0.23 | 8.64 | 0.00 | 0.00 | 0.00 | 0.00 | 0.00 | 0.03 | 0.00 | 0.00 |
| 3479000 | 7.37 | 9.65 | 0.00 | 0.00 | 0.00 | 0.00 | 0.00 | 0.10 | 0.00 | 0.00 |
| 3479000 | 0.04 | 5.33 | 0.00 | 0.00 | 0.00 | 0.00 | 0.00 | 0.00 | 0.00 | 0.00 |
| 3479000 | 0.10 | 6.10 | 0.00 | 0.00 | 0.00 | 0.00 | 0.00 | 0.00 | 0.00 | 0.00 |
| 3479000 | 0.00 | 0.51 | 0.00 | 0.00 | 0.00 | 0.00 | 0.00 | 0.00 | 0.00 | 0.00 |
| 3479000 | 0.31 | 6.35 | 0.00 | 0.00 | 0.00 | 0.00 | 0.00 | 0.00 | 0.00 | 0.00 |
| 3479000 | 1.48 | 26.16 | 0.00 | 0.00 | 0.29 | 0.00 | 0.59 | 6.04 | 0.00 | 0.59 |
| 3479000 | 0.45 | 135.13 | 2.44 | 26.73 | 62.18 | 28.56 | 65.14 | 98.08 | 2.44 | 28.56 |
| 3479000 | 0.38 | 34.29 | 0.00 | 0.00 | 1.87 | 0.00 | 2.32 | 10.94 | 0.00 | 2.32 |
| 3479000 | 0.19 | 9.40 | 0.00 | 0.00 | 0.00 | 0.00 | 0.00 | 0.08 | 0.00 | 0.00 |
| 3479000 | 1.09 | 110.24 | 0.22 | 15.21 | 43.07 | 16.70 | 45.76 | 74.74 | 0.22 | 16.70 |
| 3479000 | 0.03 | 15.49 | 0.00 | 0.00 | 0.00 | 0.00 | 0.00 | 1.34 | 0.00 | 0.00 |
| 3479000 | 0.44 | 5.08 | 0.00 | 0.00 | 0.00 | 0.00 | 0.00 | 0.00 | 0.00 | 0.00 |
| 3479000 | 0.00 | 2.54 | 0.00 | 0.00 | 0.00 | 0.00 | 0.00 | 0.00 | 0.00 | 0.00 |
| 3479000 | 2.32 | 27.69 | 0.00 | 0.00 | 0.47 | 0.00 | 0.84 | 6.89 | 0.00 | 0.84 |
| 3479000 | 0.17 | 6.10 | 0.00 | 0.00 | 0.00 | 0.00 | 0.00 | 0.00 | 0.00 | 0.00 |
| 3479000 | 0.48 | 12.45 | 0.00 | 0.00 | 0.00 | 0.00 | 0.00 | 0.54 | 0.00 | 0.00 |
| 3479000 | 0.00 | 43.43 | 0.00 | 0.00 | 4.20 | 0.01 | 5.32 | 17.31 | 0.00 | 0.01 |
| 3479000 | 0.92 | 45.72 | 0.00 | 0.00 | 4.99 | 0.06 | 6.22 | 19.00 | 0.00 | 0.06 |
| 3479000 | 0.02 | 1.78 | 0.00 | 0.00 | 0.00 | 0.00 | 0.00 | 0.00 | 0.00 | 0.00 |
| 3479000 | 0.01 | 1.02 | 0.00 | 0.00 | 0.00 | 0.00 | 0.00 | 0.00 | 0.00 | 0.00 |
| 3479000 | 0.87 | 52.07 | 0.00 | 0.19 | 7.44 | 0.46 | 8.97 | 23.88 | 0.00 | 0.46 |
| 3479000 | 0.00 | 6.35 | 0.00 | 0.00 | 0.00 | 0.00 | 0.00 | 0.00 | 0.00 | 0.00 |
| 3479000 | 0.19 | 4.83 | 0.00 | 0.00 | 0.00 | 0.00 | 0.00 | 0.00 | 0.00 | 0.00 |
| 3479000 | 0.09 | 4.57 | 0.00 | 0.00 | 0.00 | 0.00 | 0.00 | 0.00 | 0.00 | 0.00 |
| 3479000 | 1.15 | 38.35 | 0.00 | 0.00 | 2.91 | 0.00 | 3.53 | 13.68 | 0.00 | 3.53 |
| 3479000 | 29.73 | 90.93 | 0.00 | 7.58 | 28.70 | 9.22 | 31.88 | 57.09 | 7.58 | 31.88 |
| 3479000 | 0.00 | 5.84 | 0.00 | 0.00 | 0.00 | 0.00 | 0.00 | 0.00 | 0.00 | 0.00 |
| 3479000 | 0.16 | 21.59 | 0.00 | 0.00 | 0.03 | 0.00 | 0.10 | 3.73 | 0.00 | 0.10 |
| 3479000 | 1.29 | 38.35 | 0.00 | 0.00 | 2.63 | 0.00 | 3.53 | 13.68 | 0.00 | 3.53 |
| 3479000 | 0.20 | 5.33 | 0.00 | 0.00 | 0.00 | 0.00 | 0.00 | 0.00 | 0.00 | 0.00 |
| 3479000 | 0.00 | 1.52 | 0.00 | 0.00 | 0.00 | 0.00 | 0.00 | 0.00 | 0.00 | 0.00 |
| 3479000 | 2.65 | 36.58 | 0.00 | 0.00 | 2.18 | 0.00 | 2.97 | 12.46 | 0.00 | 2.97 |
| 3479000 | 0.11 | 2.03 | 0.00 | 0.00 | 0.00 | 0.00 | 0.00 | 0.00 | 0.00 | 0.00 |
| 3568933 | 16.20 | 53.09 | 0.00 | 2.80 | 14.75 | 1.17 | 11.21 | 26.73 | 2.80 | 11.21 |
| 3568933 | 7.89 | 25.15 | 0.00 | 0.00 | 1.75 | 0.00 | 0.81 | 6.45 | 0.00 | 0.81 |
| 3568933 | 10.48 | 21.34 | 0.00 | 0.00 | 0.84 | 0.00 | 0.26 | 4.37 | 0.00 | 0.26 |
| 3568933 | 5.30 | 36.58 | 0.00 | 0.21 | 5.95 | 0.00 | 3.91 | 13.93 | 0.21 | 3.91 |
| 3568933 | 16.52 | 49.53 | 0.00 | 2.02 | 12.65 | 0.70 | 9.42 | 23.84 | 2.02 | 9.42 |
| 3568933 | 3.43 | 29.97 | 0.00 | 0.00 | 3.28 | 0.00 | 1.88 | 9.42 | 0.00 | 1.88 |
| 3568933 | 8.01 | 67.56 | 0.00 | 7.06 | 24.20 | 4.09 | 19.48 | 39.01 | 7.06 | 19.48 |
| 3568933 | 3.26 | 10.16 | 0.00 | 0.00 | 0.00 | 0.00 | 0.00 | 0.31 | 0.00 | 0.00 |
| 3568933 | 5.87 | 25.65 | 0.00 | 0.00 | 1.89 | 0.00 | 0.91 | 6.75 | 0.00 | 0.91 |
| 3568933 | 2.07 | 15.75 | 0.00 | 0.00 | 0.08 | 0.00 | 0.00 | 1.87 | 0.00 | 0.00 |
| 3568933 | 4.74 | 34.29 | 0.00 | 0.08 | 4.96 | 0.00 | 3.14 | 12.32 | 0.08 | 3.14 |
| 3568933 | 2.19 | 9.40 | 0.00 | 0.00 | 0.00 | 0.00 | 0.00 | 0.19 | 0.00 | 0.00 |
| 3568933 | 0.88 | 1.27 | 0.00 | 0.00 | 0.00 | 0.00 | 0.00 | 0.00 | 0.00 | 0.00 |
| 3568933 | 3.06 | 45.72 | 0.00 | 1.31 | 10.51 | 0.33 | 7.62 | 20.81 | 1.31 | 7.62 |
| 3568933 | 1.94 | 34.54 | 0.00 | 0.09 | 5.07 | 0.00 | 3.22 | 12.49 | 0.09 | 3.22 |
| 3568933 | 2.25 | 27.94 | 0.00 | 0.00 | 2.59 | 0.00 | 1.38 | 8.13 | 0.00 | 1.38 |
| 3568933 | 0.71 | 14.22 | 0.00 | 0.00 | 0.01 | 0.00 | 0.00 | 1.34 | 0.00 | 0.00 |
| 3568933 | 1.28 | 25.91 | 0.00 | 0.00 | 1.97 | 0.00 | 0.96 | 6.90 | 0.00 | 0.96 |
| 3568933 | 4.72 | 67.06 | 0.00 | 6.89 | 23.85 | 3.97 | 19.17 | 38.57 | 6.89 | 19.17 |
| 3568933 | 0.99 | 7.62 | 0.00 | 0.00 | 0.00 | 0.00 | 0.00 | 0.02 | 0.00 | 0.00 |
| 3568933 | 1.19 | 34.80 | 0.00 | 0.10 | 5.18 | 0.00 | 3.31 | 12.67 | 0.10 | 3.31 |
| 3568933 | 0.39 | 6.60 | 0.00 | 0.00 | 0.00 | 0.00 | 0.00 | 0.00 | 0.00 | 0.00 |
| 3568933 | 0.56 | 1.27 | 0.00 | 0.00 | 0.00 | 0.00 | 0.00 | 0.00 | 0.00 | 0.00 |
| 3568933 | 0.51 | 5.59 | 0.00 | 0.00 | 0.00 | 0.00 | 0.00 | 0.00 | 0.00 | 0.00 |
| 3568933 | 0.26 | 1.02 | 0.00 | 0.00 | 0.00 | 0.00 | 0.00 | 0.00 | 0.00 | 0.00 |
| 3568933 | 0.26 | 0.51 | 0.00 | 0.00 | 0.00 | 0.00 | 0.00 | 0.00 | 0.00 | 0.00 |
| 3568933 | 0.90 | 14.73 | 0.00 | 0.00 | 0.03 | 0.00 | 0.00 | 1.51 | 0.00 | 0.00 |
| 3568933 | 1.32 | 33.27 | 0.00 | 0.04 | 4.54 | 0.00 | 2.82 | 11.62 | 0.04 | 2.82 |
| 3568933 | 0.27 | 20.83 | 0.00 | 0.00 | 0.74 | 0.00 | 0.21 | 4.11 | 0.00 | 0.21 |
| 3568933 | 1.25 | 23.11 | 0.00 | 0.00 | 1.23 | 0.00 | 0.49 | 5.31 | 0.00 | 0.49 |
| 3568933 | 0.96 | 15.75 | 0.00 | 0.00 | 0.08 | 0.00 | 0.00 | 1.87 | 0.00 | 0.00 |
| 3568933 | 0.17 | 0.25 | 0.00 | 0.00 | 0.00 | 0.00 | 0.00 | 0.00 | 0.00 | 0.00 |
| 3568933 | 0.57 | 95.50 | 2.08 | 19.10 | 45.20 | 13.59 | 38.59 | 64.18 | 2.08 | 13.59 |
| 3568933 | 0.66 | 11.68 | 0.00 | 0.00 | 0.00 | 0.00 | 0.00 | 0.62 | 0.00 | 0.00 |
| 3568933 | 0.79 | 77.22 | 0.25 | 10.72 | 31.12 | 6.86 | 25.69 | 47.54 | 0.25 | 6.86 |
| 3568933 | 0.29 | 13.21 | 0.00 | 0.00 | 0.00 | 0.00 | 0.00 | 1.02 | 0.00 | 0.00 |
| 3568933 | 0.84 | 66.04 | 0.00 | 6.54 | 23.15 | 3.71 | 18.55 | 37.69 | 0.00 | 3.71 |
| 3568933 | 0.78 | 49.78 | 0.00 | 2.07 | 12.80 | 0.73 | 9.54 | 24.04 | 2.07 | 9.54 |
| 3568933 | 0.35 | 5.59 | 0.00 | 0.00 | 0.00 | 0.00 | 0.00 | 0.00 | 0.00 | 0.00 |
| 3568933 | 1.36 | 13.97 | 0.00 | 0.00 | 0.00 | 0.00 | 0.00 | 1.26 | 0.00 | 0.00 |
| 3568933 | 0.40 | 13.21 | 0.00 | 0.00 | 0.00 | 0.00 | 0.00 | 1.02 | 0.00 | 0.00 |
| 3568933 | 3.61 | 53.85 | 0.00 | 2.99 | 15.22 | 1.28 | 11.61 | 27.35 | 2.99 | 11.61 |
| 3568933 | 1.15 | 1.52 | 0.00 | 0.00 | 0.00 | 0.00 | 0.00 | 0.00 | 0.00 | 0.00 |
| 3568933 | 1.77 | 46.99 | 0.00 | 1.53 | 11.21 | 0.44 | 8.20 | 21.81 | 1.53 | 8.20 |
| 3568933 | 2.43 | 10.92 | 0.00 | 0.00 | 0.00 | 0.00 | 0.00 | 0.45 | 0.00 | 0.00 |
| 3568933 | 2.04 | 14.73 | 0.00 | 0.00 | 0.04 | 0.00 | 0.00 | 1.51 | 0.00 | 0.00 |
| 3568933 | 1.90 | 5.08 | 0.00 | 0.00 | 0.00 | 0.00 | 0.00 | 0.00 | 0.00 | 0.00 |
| 3568933 | 0.64 | 8.89 | 0.00 | 0.00 | 0.00 | 0.00 | 0.00 | 0.12 | 0.00 | 0.00 |
| 3568933 | 3.84 | 27.43 | 0.00 | 0.00 | 2.56 | 0.00 | 1.27 | 7.81 | 0.00 | 1.27 |
| 3568933 | 1.57 | 6.86 | 0.00 | 0.00 | 0.00 | 0.00 | 0.00 | 0.00 | 0.00 | 0.00 |
| 3568933 | 1.01 | 2.29 | 0.00 | 0.00 | 0.00 | 0.00 | 0.00 | 0.00 | 0.00 | 0.00 |
| 3568933 | 6.99 | 94.49 | 2.16 | 19.15 | 45.04 | 13.17 | 37.84 | 63.25 | 2.16 | 13.17 |
| 3568933 | 3.05 | 3.05 | 0.00 | 0.00 | 0.00 | 0.00 | 0.00 | 0.00 | 0.00 | 0.00 |
| 3568933 | 12.64 | 109.98 | 5.01 | 27.53 | 57.74 | 20.06 | 49.59 | 77.67 | 5.01 | 20.06 |
| 3568933 | 8.09 | 71.37 | 0.07 | 8.79 | 27.39 | 5.12 | 21.88 | 42.35 | 8.79 | 21.88 |
| 3568933 | 2.39 | 5.33 | 0.00 | 0.00 | 0.00 | 0.00 | 0.00 | 0.00 | 0.00 | 0.00 |
| 3568933 | 1.95 | 6.35 | 0.00 | 0.00 | 0.00 | 0.00 | 0.00 | 0.00 | 0.00 | 0.00 |
| 3568933 | 0.82 | 37.85 | 0.00 | 0.36 | 6.77 | 0.00 | 4.37 | 14.85 | 0.36 | 4.37 |
| 3568933 | 1.47 | 2.54 | 0.00 | 0.00 | 0.00 | 0.00 | 0.00 | 0.00 | 0.00 | 0.00 |
| 3568933 | 0.47 | 3.56 | 0.00 | 0.00 | 0.00 | 0.00 | 0.00 | 0.00 | 0.00 | 0.00 |
| 3568933 | 0.89 | 82.04 | 0.69 | 13.21 | 35.30 | 8.46 | 28.97 | 51.88 | 0.69 | 8.46 |
| 3568933 | 4.33 | 44.20 | 0.00 | 1.18 | 9.99 | 0.22 | 6.94 | 19.62 | 1.18 | 6.94 |
| 3568933 | 1.57 | 86.36 | 1.11 | 15.19 | 38.63 | 10.00 | 31.98 | 55.80 | 1.11 | 10.00 |
| 3568933 | 0.71 | 18.80 | 0.00 | 0.00 | 0.45 | 0.00 | 0.06 | 3.14 | 0.00 | 0.06 |
| 3568933 | 0.33 | 2.03 | 0.00 | 0.00 | 0.00 | 0.00 | 0.00 | 0.00 | 0.00 | 0.00 |
| 3568933 | 0.35 | 14.22 | 0.00 | 0.00 | 0.02 | 0.00 | 0.00 | 1.34 | 0.00 | 0.00 |
| 3568933 | 1.73 | 51.56 | 0.00 | 2.63 | 14.20 | 0.95 | 10.43 | 25.48 | 2.63 | 10.43 |
| 3568933 | 0.42 | 3.05 | 0.00 | 0.00 | 0.00 | 0.00 | 0.00 | 0.00 | 0.00 | 0.00 |
| 3568933 | 2.27 | 29.72 | 0.00 | 0.00 | 3.35 | 0.00 | 1.81 | 9.25 | 0.00 | 1.81 |
| 3568933 | 1.64 | 33.78 | 0.00 | 0.08 | 4.95 | 0.00 | 2.98 | 11.97 | 0.08 | 2.98 |
| 3568933 | 0.67 | 2.54 | 0.00 | 0.00 | 0.00 | 0.00 | 0.00 | 0.00 | 0.00 | 0.00 |
| 3568933 | 0.32 | 4.06 | 0.00 | 0.00 | 0.00 | 0.00 | 0.00 | 0.00 | 0.00 | 0.00 |
| 3568933 | 0.35 | 6.10 | 0.00 | 0.00 | 0.00 | 0.00 | 0.00 | 0.00 | 0.00 | 0.00 |
| 3568933 | 0.40 | 0.51 | 0.00 | 0.00 | 0.00 | 0.00 | 0.00 | 0.00 | 0.00 | 0.00 |
| 3568933 | 4.15 | 124.46 | 8.59 | 36.16 | 70.05 | 27.38 | 61.12 | 91.35 | 8.59 | 27.38 |
| 3568933 | 2.59 | 25.91 | 0.00 | 0.00 | 2.08 | 0.00 | 0.96 | 6.90 | 0.00 | 0.96 |
| 3568933 | 2.63 | 69.09 | 0.02 | 7.94 | 25.76 | 4.49 | 20.43 | 40.34 | 0.02 | 4.49 |
| 3568933 | 2.60 | 12.19 | 0.00 | 0.00 | 0.00 | 0.00 | 0.00 | 0.74 | 0.00 | 0.00 |
| 3568933 | 1.90 | 84.07 | 0.88 | 14.13 | 36.86 | 9.17 | 30.38 | 53.72 | 0.88 | 9.17 |
| 3568933 | 1.13 | 13.97 | 0.00 | 0.00 | 0.01 | 0.00 | 0.00 | 1.26 | 0.00 | 0.00 |
| 3568933 | 0.73 | 20.32 | 0.00 | 0.00 | 0.71 | 0.00 | 0.17 | 3.86 | 0.00 | 0.17 |
| 3568933 | 2.70 | 33.78 | 0.00 | 0.08 | 4.95 | 0.00 | 2.98 | 11.97 | 0.08 | 2.98 |
| 3568933 | 1.48 | 12.19 | 0.00 | 0.00 | 0.00 | 0.00 | 0.00 | 0.74 | 0.00 | 0.00 |
| 3568933 | 1.14 | 13.72 | 0.00 | 0.00 | 0.01 | 0.00 | 0.00 | 1.18 | 0.00 | 0.00 |
| 3568933 | 0.54 | 1.27 | 0.00 | 0.00 | 0.00 | 0.00 | 0.00 | 0.00 | 0.00 | 0.00 |
| 3568933 | 1.45 | 10.67 | 0.00 | 0.00 | 0.00 | 0.00 | 0.00 | 0.40 | 0.00 | 0.00 |
| 3568933 | 0.92 | 4.57 | 0.00 | 0.00 | 0.00 | 0.00 | 0.00 | 0.00 | 0.00 | 0.00 |
| 3568933 | 0.43 | 6.60 | 0.00 | 0.00 | 0.00 | 0.00 | 0.00 | 0.00 | 0.00 | 0.00 |
| 3568933 | 0.82 | 11.43 | 0.00 | 0.00 | 0.00 | 0.00 | 0.00 | 0.56 | 0.00 | 0.00 |
| 3568933 | 0.47 | 4.57 | 0.00 | 0.00 | 0.00 | 0.00 | 0.00 | 0.00 | 0.00 | 0.00 |
| 3568933 | 0.84 | 16.00 | 0.00 | 0.00 | 0.12 | 0.00 | 0.00 | 1.97 | 0.00 | 0.00 |
| 3568933 | 1.08 | 18.54 | 0.00 | 0.00 | 0.41 | 0.00 | 0.05 | 3.03 | 0.00 | 0.05 |
| 3568933 | 3.58 | 25.91 | 0.00 | 0.00 | 2.08 | 0.00 | 0.96 | 6.90 | 0.00 | 0.96 |
| 3568933 | 2.20 | 60.71 | 0.00 | 5.11 | 20.00 | 2.51 | 15.39 | 33.10 | 5.11 | 15.39 |
